# A Systematic Review of On-Site Rapid Detection Methods for Antibiotic Residues in Aquatic Products (2021–2025)

**DOI:** 10.3390/foods15071264

**Published:** 2026-04-07

**Authors:** Guangyao Ying, Tingting Wang, Kunlun Li, Yuxin Wang, Jinjun Zhang, Gangjian Lin, Jun Li, Huili Xia, Jinjie Zhang, Liang Hong

**Affiliations:** 1 Taizhou Institute for Food and Drug Control, Taizhou 318000, China; tzsyygy@126.com (G.Y.);; 2 Taizhou Key Laboratory of Model Animal and Preclinical Pharmaceutical Research, Taizhou 318000, China; 3 Key Laboratory for Quality Safety and Quality Improvement of Characteristic Agricultural Products in Taizhou City, Taizhou 318000, China; 4 Taizhou Center for Disease Control and Prevention, Taizhou 318000, China; wja_wdt@163.com; 5 School of Pharmaceutical Sciences, Jinhua Academy, Zhejiang Chinese Medical University, Hangzhou 310053, China; 6 School of Food Science and Engineering, Ningbo University, Ningbo 315211, China

**Keywords:** aquatic products, antibiotic residues, on-site rapid detection, fluorescent sensor, lateral flow immunoassay, SERS, ELISA, electrochemical sensor, colorimetric sensor

## Abstract

Antibiotic residues in aquatic products pose a serious food safety concern, whereas conventional laboratory methods often fail to meet the demand for on-site rapid screening. This study systematically reviews the research progress from 2021 to 2025 on both the risks of antibiotic residues in aquatic products and the development of rapid on-site detection technologies. First, based on a literature survey covering major aquatic products (e.g., fish, shrimp, and shellfish), the widespread occurrence of multiple antibiotics at high concentrations was documented, with quinolones and sulfonamides identified as the most frequently detected classes. To address the need for on-site testing, this review focuses on six rapid detection techniques: fluorescent sensor (FRS), lateral flow immunoassay (LFIA), surface-enhanced Raman scattering (SERS), enzyme-linked immunosorbent assay (ELISA), electrochemical sensor (ECRS), and colorimetric sensor (CRS). The core principles, technical advantages, recent application cases (e.g., integration with smartphones and novel nanomaterials), and development trends for each method are analyzed. Finally, it discusses the current challenges faced by existing on-site detection approaches and their potential solutions. Technology selection strategies tailored to different application scenarios (e.g., aquaculture farms, distribution channels, and consumer-level use) are also proposed.

## 1. Introduction

The invention and application of antibiotics represent a milestone in modern medicine and agricultural production, having made indelible contributions to safeguarding human and animal health and promoting the development of aquaculture [1]. However, their extensive and often irrational use has led to severe issues of residue accumulation and antimicrobial resistance, posing an ongoing threat to global public health security [2]. In aquaculture, antibiotics are frequently used not only as therapeutic agents but also as growth promoters to prevent and control bacterial diseases, thereby improving survival rates and yield in intensive farming systems [3]. Nevertheless, non-standard practices such as off-label use, overdose, or failure to observe withdrawal periods directly result in the accumulation of antibiotics and their metabolites in aquatic organisms [4]. These drug residues pose direct risks to consumer health, including allergic reactions and disruption of gut microbiota. More critically, long-term, low-dose exposure can accelerate the selection and spread of resistant bacterial strains in humans. This may ultimately lead to a future where effective treatments for bacterial infections are unavailable [5]. Therefore, establishing an efficient and reliable monitoring system for antibiotic residues is a crucial line of defense to ensure the quality and safety of aquatic products and protecting consumer health.

To address these risks, the development of effective detection methods is a prerequisite for robust regulatory oversight. Currently, confirmatory laboratory-based methods, such as high-performance liquid chromatography-tandem mass spectrometry (HPLC-MS/MS), are considered the gold standard due to their high sensitivity, accuracy, and their capability for simultaneous identification and quantification of multiple compounds [6]. However, these methods rely on expensive, large-scale instrumentation, complex sample preparation procedures, specialized personnel, and lengthy analysis times. Consequently, they are ill-suited to meet the urgent need for the rapid, on-site, and low-cost screening of large sample batches in settings such as farms, wholesale markets, and customs checkpoints [7]. The time lag between sampling and reporting means that contaminated products may have already entered the market by the time results are available, undermining pre-market risk interception efforts. Hence, the development of rapid, sensitive, user-friendly, cost-effective, and field-deployable detection technologies has become a major research focus and an inevitable trend in food safety testing.

In recent years, the convergence of nanotechnology, materials science, immunology, and microelectronics has given rise to a series of novel on-site rapid detection technologies, offering promising solutions to these challenges. These technologies aim to “transfer” or “simplify” complex laboratory analytical processes into portable devices or even test strips, enabling a “sample-in, answer-out” rapid detection mode. Among these, FRS, LFIA, SERS, ELISA, ECRS, and CRS have garnered significant research attention due to their respective advantages in sensitivity, specificity, detection speed, portability, and/or cost-effectiveness. For instance, smartphone-integrated FRS can achieve visual quantitative detection [8]; LFIA test strips can complete screening within 10 min [9]; and SERS technology can provide a substance-specific “fingerprint” spectrum with high specificity [10]. These technologies are rapidly evolving towards higher sensitivity, multiplexing capability, enhanced robustness against interference, and greater integration and intelligence (e.g., integration with the Internet of Things).

Nevertheless, the successful application of these advanced sensing technologies to the on-site detection of antibiotics in complex matrices such as aquatic products still faces numerous challenges. Abundant proteins, fats, and other components matrix components can severely interfere with the detection signal [11]. The diverse physicochemical properties of different antibiotics require methods to possess either broad universality or high specificity [12]. Furthermore, the transition from high laboratory performance to stable and reliable operation in field environments requires further breakthroughs in sensor stability, batch-to-batch reproducibility, cost control, and ease of operation [13]. Therefore, a systematic review of the principles, recent advances, performance comparisons, and scenario-specific suitability of different rapid detection technologies is of significant importance for guiding the transition of this field from laboratory research to practical application.

Compared to previous reviews, this work addresses the following critical gaps. First, it systematically summarizes research progress from 2021 to 2025, ensuring content timeliness. Second, it focuses specifically on aquatic products as a complex and challenging matrix, detailing their sample pretreatment challenges and the consequent specific requirements for detection technologies. More importantly, this review goes beyond a descriptive introduction. It provides a comparative analysis (as summarized in Table 1) of the core performance parameters of the six key technologies. Furthermore, it integrates technical performance with the demands of specific application scenarios, such as aquaculture, distribution, and consumption, to propose guided technology selection strategies. Therefore, this review not only provides researchers with a comprehensive overview of the latest technological developments but also serves as a practical reference for practitioners in selecting appropriate on-site detection tools for different stages of the supply chain.

Accordingly, this review aims to systematically elaborate on the current status and risks of antibiotic residues in aquatic products, with a primary focus on the six aforementioned on-site rapid detection technologies. This review first analyzes the overall occurrence and high-risk antibiotics based on literature from 2021 to 2025. It then provides an in-depth exploration of the core mechanisms, performance optimization strategies (e.g., using novel nanomaterials, signal amplification, smartphone integration), practical application cases, and associated challenges for each rapid detection technology. Finally, it discusses technology selection strategies for different supervision scenarios (e.g., farming sources, circulation points, and consumer endpoints) and provides an outlook on future development directions. The goal is to provide a reference for the development of more efficient and practical on-site rapid detection technologies, thereby contributing to the establishment of a smart, full-chain food safety supervision network “from pond to table”.

To facilitate reading, the abbreviations, full names, and classifications of antibiotics involved in this study are listed in Table 2.

## 2. Literature Search Process

**Figure 1:**
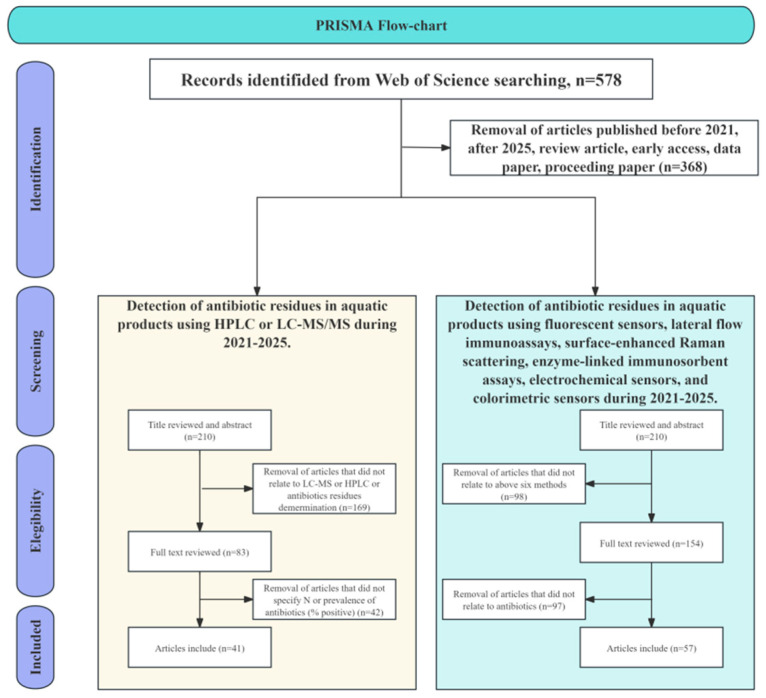
Scheme of the adopted PRISMA flow-chart.

The specific workflow of the literature search and screening process is summarized in the PRISMA flow diagram (Figure 1). As illustrated, a dual-path screening strategy was adopted: the left path focused on studies that used high-performance liquid chromatography (HPLC) or liquid chromatography-tandem mass spectrometry (LC-MS/MS) to detect antibiotic residues in aquatic products, aiming to clarify the background residue profile; the right path systematically screened the literature on six emerging on-site rapid detection technologies, including FRS and LFIA. Initially, 578 relevant records were retrieved from the Web of Science database using the search query: TS = (“aquatic product” OR seafood) AND TS = (“antibiotic residue” OR “veterinary drug residue”). After applying filters for publication date (2021–2025) and document type (excluding reviews, early access, data papers, and conference proceedings), and after an initial title/abstract screening (n = 210), full-text assessment was conducted (left path: n = 83; right path: n = 154). This was followed by further refinement based on strict inclusion and exclusion criteria. Ultimately, 98 articles were included in this review: 41 articles were used to elucidate the profile of antibiotic residues based on HPLC or LC-MS/MS methods, and 57 articles were used for the in-depth analysis and comparison of emerging on-site rapid detection technologies. The full details of all 57 studies focusing on rapid detection technologies are provided in a searchable database as Supplementary Material Table S1.

## 3. Risks of Antibiotic Residues in Common Aquatic Products

Studies have indicated that the extensive and improper use of antibiotics leads to their introduction into aquatic environments via various pathways, resulting in the accumulation of significant antibiotic residues in the tissues of aquatic organisms [69]. The consumption of such antibiotic-contaminated aquatic products may pose serious health risks to consumers [70]. This study reviewed monitoring studies on antibiotic residues in various aquatic products published in the Web of Science database between 2021 and 2025, that employed chromatographic or mass spectrometric methods. The findings are summarized by category (fish, shrimp, shellfish, crab, frog, and echinoderms) in Table 3. Fish were found to be the category with the highest number of different antibiotic residues detected, with 49 distinct antibiotics identified. This was followed by shrimp and crabs, each with 21 different antibiotics detected. Echinoderms had 5 antibiotics detected, whereas frogs had the fewest, with only 1 antibiotic detected.

### 3.1. Fish

Studies indicate that following antibiotic administration in aquaculture, residue levels in fish muscle tissue at various sampling intervals can exceed the maximum residue limits (MRLs) established by regulatory authorities [111]. Although most antibiotic residues fall below the MRLs after an appropriate withdrawal period, residues of enrofloxacin [112], trimethoprim [113], and oxytetracycline [114] have been reported to persist above the permissible levels. Furthermore, common cooking methods such as boiling, frying, and baking are not effective in eliminating these antibiotic residues [115,116]. Therefore, monitoring antibiotic residues in fish is crucial for ensuring consumer safety.

This review analyzed 31 studies on antibiotic residues in fish published between 2021 and 2025, as summarized in Table 3. A total of 49 different antibiotic residues were identified in fish samples. Quinolones were the most frequently detected class (15 compounds), followed by sulfonamides (10 compounds). The antibiotics with the highest detection frequencies were CIP, ENR, DOX, OTC, TMP, OFX, SDZ, SMX, and SMZ. CIP was reported in 13 studies. The highest residue level (113.79 mg/kg) was detected in fish liver from a farm in Lagos, Nigeria [71], exceeding the EU limit by more than 1100 times [117]. Similarly, ENR was also reported in 13 studies, with the highest residue level (9.61 ppm) found in China [85], exceeding the EU limit by 96.1 times [117]. These findings indicate that the situation regarding antibiotic residues in fish remains serious, characterized by the co-occurrence of multiple antibiotics at high concentrations. Relying solely on post-market sampling and laboratory instrumental analysis is insufficient to address the problem of antibiotic misuse in aquaculture. Therefore, there is a clear need for on-site rapid screening technologies that can be applied at the initial stages of the supply chain (e.g., at the farm level).

### 3.2. Shrimp

Compared to fish, the situation regarding antibiotic residues in shrimp appears to be less severe. As shown in Table 3 residues of only 19 antibiotics across 5 major classes were reported in 10 studies between 2021 and 2025. This difference may be attributed to variations in organs for antibiotic accumulation, farming practices, and trophic levels between shrimp and fish [118]. Nevertheless, sulfonamides and quinolones remained the predominant classes of residues detected in shrimp, accounting for 68.4% of the total. The highest residue level reported was for ENR at 3.683 ppm [104], which is 36.83 times the EU limit [119]. Another factor contributing to the relatively lower perceived risk from shrimp may be related to the parts tested or consumed. The hepatopancreas in the shrimp head can retain higher levels of antibiotics for longer periods [120], whereas testing and consumption typically focus only on the muscle tissue. However, sulfonamide drugs tend to accumulate in muscle tissue [121], which may explain why they are the most frequently detected class in shrimp.

### 3.3. Other Categories of Aquatic Products

Research on antibiotic residues in crabs, shellfish, frogs, and echinoderms is less extensive than that on fish and shrimp. One possible reason is their smaller scale of farming and consumption. For instance, frogs and echinoderms are widely consumed only in specific regions, which may result in less regulatory attention and consequently fewer related studies [122,123]. Another reason may be a perceived lower risk of antibiotic contamination. For example, crabs are often farmed at lower densities than fish and shrimp, potentially leading to less reliance on antibiotics during cultivation [124,125]. Furthermore, The feed used for shellfish and echinoderms often differs from that for fish and shrimp, which may present a lower risk of antibiotic introduction from feed sources [126,127]. As indicated in Table 3 only 7 studies reported the detection of antibiotics in crabs, shellfish, frogs, and echinoderms between 2021 and 2025. Only CTC in shellfish [94] and ENR in frogs [85] were found to substantially exceed regulatory limits. Antibiotics detected in crabs and echinoderms were predominantly within safe limits. In summary, while antibiotic residues in fish and shrimp pose a significant concern, the situation in other aquatic product categories appears less severe. However, monitoring for several commonly used antibiotics, such as quinolones, sulfonamides, and tetracyclines, remains necessary across all categories.

Analysis of the data summarized in Table 3 reveals several clear trends. During the 2021–2025 period, quinolones (e.g., enrofloxacin, ciprofloxacin) and sulfonamides (e.g., sulfamethoxazole, sulfadiazine) are the most frequently detected classes of antibiotic residues across various aquatic products, especially in farmed fish and shrimp. Tetracyclines and macrolides are also commonly reported. Notably, some studies have reported residue concentrations of antibiotics such as sulfamethoxazole, enrofloxacin, and chloramphenicol exceeding the maximum residue limits (MRLs) set by standards in China, the EU, or Codex in specific regions and products. These data underscore the continued presence and potential health risks posed by antibiotic residues in aquatic products. In summary, given the widespread occurrence and high-concentration residues of antibiotics in various aquatic products, relying solely on conventional laboratory instruments like HPLC or LC-MS for routine monitoring presents significant challenges and cannot fundamentally solve the problems of antibiotic abuse and residue. Therefore, it is imperative to employ on-site detection technologies for monitoring at the source of the aquatic product supply chain (e.g., farms, processing plants, wholesale markets). This approach can effectively reduce the risk of antibiotic residues entering the human food chain.

## 4. Methods for On-Site Antibiotic Detection

**Figure 2:**
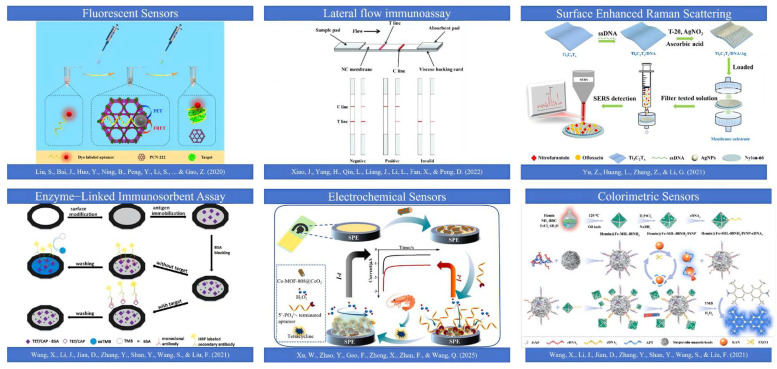
Schematic diagram of the principles of the six main on-site rapid detection methods [10,21,37,47,62].

**Figure 3:**
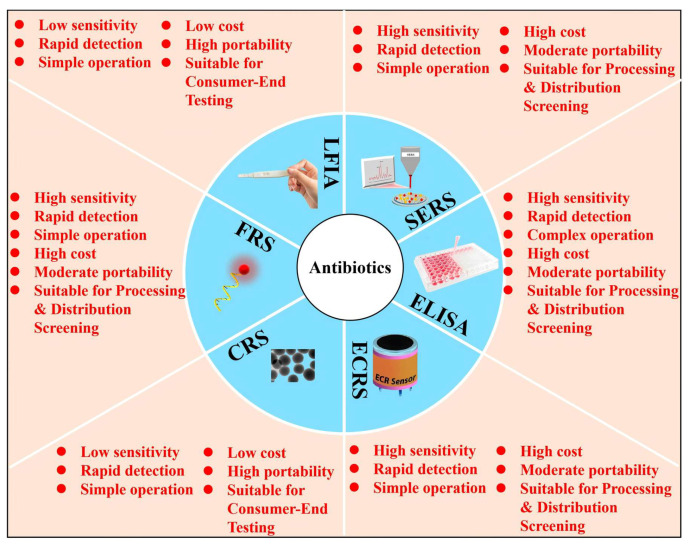
Overview of on-site rapid detection methods f or antibiotic residues and their performance.

Based on a review of reported studies on the on-site rapid detection of other small-molecule contaminants (such as mycotoxins and pesticide residues) [128,129], six methods were identified six methods, CRS, FRS, ELISA, LFIA (test strips), SERS, and ECRS, as being more suitable for the on-site rapid detection of antibiotics in aquatic products. This assessment was based on key factors for field deployment, including sample pretreatment requirements, detection workflow complexity, device cost-effectiveness, and overall feasibility. Consequently, a targeted search was conducted in the Web of Science database using keywords encompassing the major aquatic products (fish, shrimp, shellfish, crab, frog, echinoderms), the six detection methods, and antibiotics, covering the period from 2021 to 2025. From the initial search results, 57 studies were identified as being directly related to rapid antibiotic detection methods in aquatic products. The technology type, sample matrix, antibiotic(s) detected, core techniques and materials involved, and performance parameters of these studies are summarized in Table 1 FRS was the most popular, accounting for 26.3% of the studies. This was followed by LFIA, SERS, ELISA, and ECRS, with 11, 10, 9, and 8 studies, respectively. CRS was the least represented, with only 4 studies in the past five years. Schematic diagrams illustrating the working principles of these six detection methods are presented in Figure 2 and the subsequent sections, and a comparative summary of their performance is provided in Figure 3 and Table 1.

### 4.1. FRS

**Figure 4:**
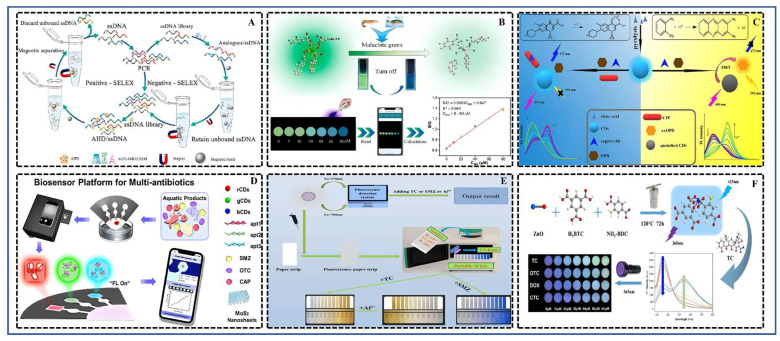
Working principle of FRS for detecting antibiotic residues in aquatic products. (**A**): Schematic diagram of aptamer selection based on MCP−SELEX for AHD [56]. (**B**): Schematic diagram of a Tb−MOF-based agar-slice detection platform combined with a smartphone for visual detection of MG in aquatic products [8]. (**C**): The ratiometric fluorescent strategy for detection of Cu2+ and CIP [68]. (**D**): Simultaneous Visual Detection of Multiple Antibiotics via the mCD−μPAD Aptasensor and a Portable Detection Device with a Smartphone [60]. (**E**): D/U−CTCRFS for the detection of TC, SMZ and cascade detection of Al3+ and smartphone with paper strip based on D/U−CTCRFS for the visual detection of TC, SMZ and cascade visual detection of Al3+ [61]. (**F**): Preparation Process and Application Effect of the ZTD Fluorescence Sensor [66].

FRS exhibit significant potential in food safety detection, particularly for analyzing harmful residues like antibiotics and pesticides, owing to their advantages of high sensitivity, rapid response, and ease of operation. Their core detection principle relies on changes in fluorescence signals induced by specific interactions between the target analyte and a designed fluorescent probe. Specific mechanisms include, but are not limited to, the inner filter effect (IFE), photoinduced electron transfer (PET), Fluorescence resonance energy transfer (FRET), and conformation switching based on specific recognition (e.g., by aptamers) [130,131]. These interactions lead to measurable fluorescence quenching or enhancement, enabling the qualitative and quantitative analysis of the target. Compared to traditional chromatographic-mass spectrometric methods, fluorescence sensing offers distinct advantages: (1) High sensitivity and low detection limits (LODs), with many studies reporting LODs at the parts-per-trillion (ppt) level [62]; (2) Good selectivity, achieved through rational probe design or the incorporation of specific recognition elements like aptamers [56] or molecularly imprinted polymers [104], which can effectively distinguish structural analogues; (3) Rapid and simple operation, often requiring minimal sample preparation and featuring short response times; (4) Low cost and ease of miniaturization, especially when integrated with smartphone imaging technology, enabling real-time, on-site detection [8]. The schematic diagram illustrating the principles of both single and multiplex detection of antibiotic residues in aquatic products using FRS was shown in Figure 4.

Recent research has led to the development of various high-performance fluorescence sensing platforms. Metal/heterometallic organic frameworks (MOFs/UOFs) are widely used due to their tunable structures and luminescent properties. For example, Yang et al. [57] synthesized three heterometallic uranyl organic frameworks (UOFs) via a two-step dissolution-recrystallization structural transformation (DRST) strategy by controlling reaction time. Among them, compound 3 served as a multifunctional fluorescent probe. Through mechanisms like IFE and PET, it exhibited fluorescence quenching responses to tetracycline antibiotics and metronidazole, and a fluorescence enhancement response to the pesticide tebuconazole. The LODs for doxycycline, metronidazole, and tebuconazole were 2.385 μM, 1.465 μM, and 1.091 μM, respectively. This platform was further integrated with smartphone RGB (Red, Green, Blue) analysis, enabling visual, quantitative detection of multiple contaminants in real samples (pork, honey, fruits, and vegetables) with satisfactory recovery rates. Similarly, a zirconium-porphyrin MOF (PCN-222) was constructed as a ratiometric fluorescent biosensor. Through a competitive interaction leading to fluorescence quenching, it was used to detect chloramphenicol with an LOD as low as 0.08 pg mL^−1^ [62]. Another study utilized a Tb(III)-based supramolecular framework, where changes in the ligand-to-metal energy transfer (LMET) efficiency upon framework binding with metronidazole enabled highly selective detection of metronidazole with an LOD of 0.16 μM [58].

Covalent organic frameworks (COFs), with their high stability, designable pores, and excellent optical properties, have also become a research hotspot. Wang et al. [59] developed a phenylboronic acid-functionalized fluorescent COF (BA@COF). It could specifically recognize and quench the fluorescence of rifamycin antibiotics via hydrogen bonding and electrostatic interactions, while simultaneously exhibiting a sensitive fluorescence enhancement response to water content in ethanol, realizing “one material, dual functions” sensing. Li et al. [66] reported a dual-ligand zinc-based MOF for the ratiometric and visual fluorescence detection of tetracycline. Its dual-emission characteristics effectively mitigated environmental interference.

Aptasensors combine the high specificity of nucleic acid aptamers with the high sensitivity of fluorescent signal output. Zheng et al. [56] employed magnetic cross-linking precipitation (MCP)-SELEX technology to efficiently screen a high-affinity aptamer (Kd = 86 nM) for the nitrofurantoin metabolite 1-aminohydantoin. Based on this aptamer and the fluorescence quenching effect of graphene oxide (GO), they constructed an aptasensor for AHD with a linear range of 0.25–150 ng/mL and an LOD as low as 0.2 ng/mL, demonstrating good recovery rates in various animal-derived foods. Ding et al. [67] developed a FRET-based “turn-on” fluorescent aptasensor for the ultrasensitive detection of enrofloxacin. This sensor employed aptamer-modified core–shell upconversion nanoparticles (UCNPs) as the fluorescence donor and the metal–organic framework MIL-101(Cr) as the fluorescence quencher (i.e., energy acceptor). The operating principle was based on the spectral overlap between the fluorescence emission of UCNPs and the absorption of MIL-101(Cr), and their proximity via π-π stacking interactions, which led to the quenching of UCNPs fluorescence. Upon the introduction of enrofloxacin, the aptamer preferentially bound to ENR, underwent a conformational change, and detached from the surface of MIL-101(Cr), resulting in the recovery of the UCNPs fluorescence. The sensor achieved a LOD as low as 0.034 ng·mL^−1^, exhibited a good linear relationship in the range of 0.1 to 1000 ng·mL^−1^, and was successfully applied to detect enrofloxacin in real fish and shrimp samples.

Ratiometric and visual sensors improve accuracy through built-in reference signals or color changes, enabling naked-eye detection. Gao et al. [68] constructed a ratiometric fluorescent platform based on carbon dots (CDs) and copper ions (Cu^2+^). The coordination of Cu^2+^ with ciprofloxacin restored red fluorescence, while the blue fluorescence of CDs served as an internal reference, enabling the highly selective detection of ciprofloxacin and Cu^2+^. Tang et al. [61] developed a portable three-color ratiometric fluorescent paper sensor. By loading different fluorescent probes onto paper strips, it achieved the intelligent visual discrimination of multiple antibiotics and Al^3+^. Song et al. [8] embedded Tb-MOF into agarose slices to create a portable probe. Combined with a smartphone, it enabled rapid, on-site fluorescence detection of malachite green in aquatic products. Tong et al. [60] integrated multicolor fluorescent carbon dots on a paper-based microfluidic chip, achieving the simultaneous visual detection of sulfamethazine, oxytetracycline, and chloramphenicol.

In summary, current trends in fluorescent sensing technology development include: (1) Multifunctional and array-based sensing: A single sensor detects multiple targets simultaneously or combines sensing with adsorption functions, improving analysis throughput and material utilization. (2) Portability and intelligence: Deep integration with paper-based devices and smartphones significantly promotes the transition from laboratory analysis to on-site rapid detection. (3) Deep integration of recognition mechanisms: Combining the structural designability of MOFs/COFs with the biological specificity of aptamers, or utilizing ratiometric signal output modes, are effective strategies for improving selectivity and anti-interference capability. However, future research must address several challenges, including signal interference from complex food matrices, the long-term stability and reusability of sensory elements, and the need for simplified, low-cost fabrication processes to facilitate large-scale deployment in real-world regulatory scenarios.

Overall, FRS offer significant advantages for trace analysis requiring ultra-low detection limits, owing to their high sensitivity and quantitative output signals. However, the frequent reliance on specialized and costly detection equipment can hinder portability and limit widespread on-site deployment, reducing cost-competitiveness and operational simplicity compared to techniques such as LFIA. Additionally, potential interference from matrix autofluorescence in real aquatic product samples remains a challenge for achieving robust selectivity in complex matrices, necessitating careful probe design and sample pretreatment.

### 4.2. LFIA

**Figure 5:**
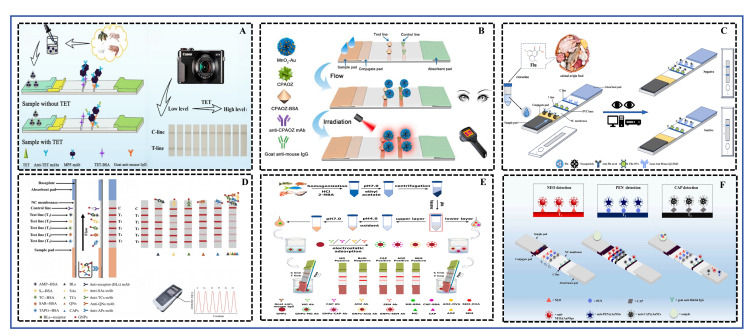
Working principle of LFIA for detecting antibiotic residues in aquatic products. (**A**): The detection of TET by MPF−LFA sensor and the interpretation of the assay results [45]. (**B**): Schematic illustration of the dual-signal immunoassay based on MnO_2_−Au for AOZ detection [50]. (**C**): Schematic illustration of the LFIS method for Flu detection [52]. (**D**): Schematic and illustration of the multiplex immunochromatographic strip and handheld reader [48]. (**E**): Schematic diagram of ICA for multi−class antibiotic residue detection [54]. (**F**): Schematic diagram of MICS for simultaneous detection of NEO, PEN, and CAP [44].

LFIA, also known as an test strip, is a rapid detection technique based on the specific antigen–antibody reaction. Its core detection principle typically employs a competitive format. The sample solution is applied to the sample pad and flows along the strip via capillary action. As it passes the conjugate pad, target analytes (e.g., antibiotic molecules) in the sample bind to specific antibodies pre-immobilized on nanomaterial signal labels (e.g., colloidal gold). This complex continues to flow to the test line (T line), where an antigen analogue (coating antigen) of the target is fixed. If the sample contains no or a low concentration of the target, the labeled antibodies can bind extensively to the antigen on the T line, forming a clear visible signal (e.g., a red band). Conversely, if the sample contains a high target concentration, it competitively occupies the antibody binding sites, leading to a weakened or absent T line signal. The control line (C line) is coated with a secondary antibody that captures any remaining labeled antibody, ensuring the validity of the test process [46,47]. This principle ensures detection specificity. The schematic diagram illustrating the principles of both single and multiplex detection of antibiotic residues in aquatic products using LFIA was shown in Figure 5.

The main advantages of LFIA lie in its speed, simplicity, low cost, and independence from complex instruments, making it highly suitable for on-site screening and large-scale preliminary testing [132]. Compared to confirmatory methods like LC-MS/MS, LFIA can reduce detection time from several hours to approximately 10 min [9]. Additionally, test strips are generally stable and can be stored at room temperature for extended periods, facilitating transportation and distribution. Although the sensitivity of traditional colloidal gold-based LFIA may be inferior to that of laboratory methods, it has been greatly improved through innovations in signal labels and detection strategies, enabling LODs in real samples that meet or approach regulatory requirements. In recent years, considerable research has focused on enhancing the sensitivity, specificity, and multiplexing capability of LFIA by developing novel nano-signal labels and optimizing detection modes.

Regarding sensitivity enhancement and multiplex detection, researchers have employed various nanomaterial strategies. For example, Cao et al. [54] developed a Au nanoparticle (AuNP)-based LFIA combined with rapid sample pretreatment, achieving sensitive detection of multiple classes of antibiotic residues in aquatic products, with LODs for four antibiotics like Chloramphenicol as low as 0.01 μg/kg. Lei et al. [48] utilized AuNPs to achieve simultaneous detection of 83 antibiotics, demonstrating the great potential of LFIA for high-throughput screening. To enable parallel, differentiated detection of multiple targets, Arsawiset et al. [44] constructed a multicolor AuNP-based LFIA that could simultaneously and visually decode the presence of neomycin, penicillin, and chloramphenicol via differently colored bands. These studies show that with rational design of antibody pools and preparation of nanoprobes, LFIA is fully capable of rapid screening for multiple residues in complex matrices, and multiplexing strategies are key to improving on-site detection efficiency.

In terms of signal amplification and diversified readout strategies, the introduction of novel nanomaterials has significantly enhanced performance. Zhong et al. [46] utilized the zirconium-based porphyrin metal–organic framework (PCN-222), which possesses both chromogenic and red fluorescent properties, to construct a dual-signal output LFIA for detecting a furazolidone metabolite. The visual LOD of the fluorescence mode (0.5 ng/mL) was 10 times lower than that of the traditional colloidal gold method (5 ng/mL). Tian et al. [45] employed a metal-polydopamine framework as a label to achieve highly sensitive detection of tetracycline with an LOD of 0.045 ng/mL. Su et al. [50] developed a dual-signal (colorimetric/photothermal) LFIA based on MnO_2_-Au composite nanoflowers for detecting furazolidone. The photothermal readout mode lowered the visual LOD from 1 ng/mL to 0.43 ng/mL. This study posits that such “dual-signal” or “multi-mode” readout strategies not only provide a built-in cross-verification mechanism, enhancing result reliability, but more importantly, they break the sensitivity bottleneck of visual judgment by introducing instrument-assisted readouts (e.g., portable thermal imagers) without substantially increasing costs. This represents an important developmental path for future high-sensitivity on-site detection.

Regarding coping with complex sample matrices and expanding application scope, functionalized nanomaterials also show advantages. Huang et al. [53] combined liposome-modified PCN-222 with a microfluidic chip to develop a “chip-LFIA” dual-model for sulfonamide antibiotic detection, effectively improving detection stability and automation potential. Liu et al. [52] developed a highly sensitive and specific nanoparticle-based LFIA for flumequine. For detecting nitrofuran metabolites, Mei et al. [49] used europium nanoparticles to achieve simultaneous fluorescence detection of three metabolites, while Teepoo et al. [51] utilized highly sensitive branched gold nanoblackbody plasmonic materials to detect leucomalachite green in fish and shrimp. These studies indicate that “tailoring” functionalized nanoprobes for specific targets and sample matrices (e.g., aquatic products, meat) is an effective means of solving matrix interference in real samples and improving method applicability and accuracy.

In summary, as a mature rapid detection platform, the development of LFIA increasingly relies on the intersection of materials science, immunology, and microfluidics. Current research continuously advances LFIA in terms of sensitivity, multiplexing capability, and result reliability by innovating signal labels (e.g., multifunctional MOFs, specially shaped metal nanostructures, rare-earth-doped particles) and designing diverse readout strategies. In the future, with the development of more high-performance, low-cost nanomaterials and deeper integration with mobile terminals like smartphones, LFIA is expected to play a more precise and powerful role in on-site, point-of-care testing across fields such as food safety, environmental monitoring, and clinical diagnostics.

Overall, LFIA has become the most mature and widely adopted technology for on-site screening due to its instrumental simplicity, rapid operation (typically 10–15 min), and low cost. However, its quantitative capability is relatively weak, its sensitivity is generally lower than that of laboratory methods, and visual result interpretation can be subjective. Compared to ECRS that provide precise digital readouts or smartphone-assisted CRS, LFIA is less suited for detailed data recording and traceability. Furthermore, its performance is highly dependent on antibody quality, and it may face challenges such as inter-band interference in multiplex detection formats.

### 4.3. SERS

**Figure 6:**
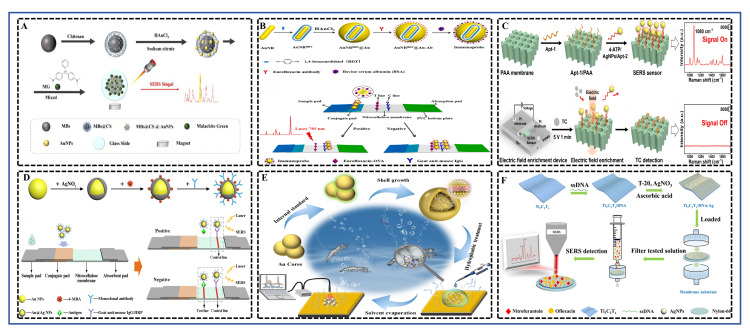
Working principle of SERS for detecting antibiotic residues in aquatic products. (**A**): Schematic diagram of the principle of the SERS substrate based on MBs@CS@AuNPs for detecting MG in aquatic products [31]. (**B**): Schematic illustration of the preparation of immunoprobe (AuNRBDT@Au−Ab) and assembly of LFIA strip and the principle of competitive SERS−LFIA for ENR [32]. (**C**): Fabrication and Schematic Illustrations of the SERS Sensor Combined with Electric Field Enrichment Technology for TC Rapid Detection [35]. (**D**): Schematic diagram of the LFA platform coupled to Au@Ag NPs and SERS for detection of CAP, TAP, and FFC [28]. (**E**): Schematic diagram showing the principle of highly sensitive SERS detection of NFL in shrimp based on Au@IS@Au NSs [34]. (**F**): Schematic diagram illustrating the principle of highly sensitive SERS detection of NFT and OFX in aquatic products based on Ti3C2Tx/DNA/Ag Membrane Substrate [10].

SERS is a powerful technique for trace analysis. Its detection principle is primarily based on two enhancement mechanisms: electromagnetic enhancement (EM) and chemical enhancement (CM) [10]. Electromagnetic enhancement originates from the localized surface plasmon resonance generated by noble metal nanostructures (e.g., gold, silver) upon laser excitation, creating intense localized electromagnetic fields, known as “hot spots”, at nanogaps or sharp edges. This can amplify the Raman signal of nearby molecules by up to 10^6^–10^8^ fold [133]. Chemical enhancement involves charge transfer between the analyte molecule and the substrate material, altering the molecular polarizability and thereby further enhancing its Raman signal [134]. The schematic diagram illustrating the principles of both single and multiplex detection of antibiotic residues in aquatic products using SERS was shown in Figure 6.

Compared to traditional methods like high-performance liquid chromatography (HPLC) or microbiological assays, SERS technology offers distinct advantages. Firstly, it exhibits extremely high sensitivity, enabling trace detection at the μg/kg or even ng/kg level [35]. Secondly, SERS provides molecular “fingerprint” spectra with strong specificity and minimal interference from water molecules, making it highly suitable for the direct or rapid analysis of complex matrices (e.g., aquatic products, food) after simple pretreatment [135]. Furthermore, SERS instruments can be miniaturized, facilitating on-site rapid screening.

Recently, researchers have designed various high-performance SERS substrates and detection strategies to improve practicality. Yu et al. [30] prepared a magnetic Ti_3_C_2_T_x_/Fe_3_O_4_/Ag composite substrate. Utilizing the large specific surface area and strong adsorption capacity of Ti_3_C_2_T_x_ MXene material, combined with the magnetic separation property of Fe_3_O_4_ and the electromagnetic enhancement from silver nanoparticles (AgNPs), they achieved highly selective enrichment and detection of sulfonamide drugs (e.g., phthalylsulfathiazole and silver sulfadiazine) in aquatic products. The LODs were 26.8 μg/L and 16.9 μg/L, respectively, with recoveries of 80.2–116% in real samples. The same team [10] also developed a Ti_3_C_2_T_x_/DNA/Ag membrane substrate, using single-stranded DNA as a template to guide the uniform growth of AgNPs loaded onto a nylon 66 membrane. This substrate was successfully used for the simultaneous quantitative detection of nitrofurantoin and ofloxacin in aquatic products, with LODs of 12.0 μg/L and 35.0 μg/L, respectively, and recoveries of 88.0–107%, demonstrating excellent anti-interference capability and potential for multi-target synchronous analysis. Yang et al. [31] prepared chitosan-functionalized magnetic gold nanoparticles (MBs@CS@AuNPs). Utilizing their magnetic and enrichment capabilities combined with a portable Raman spectrometer, they achieved rapid detection of malachite green in aquatic products with an LOD of 10^−9^ M. Huang et al. [34] synthesized gold@internal standard@gold nanospheres (Au@IS@Au NSs) with a core–shell-gap structure. The study embedded different thiol molecules (e.g., BDT) as internal standards (IS) into the nanogap between the gold core and gold shell to construct a SERS internal standard substrate with high stability and reproducibility. This substrate, combined with a hydrophobically modified gold film, utilized the hydrophobic effect during solvent evaporation to concentrate target molecules and generate additional “hot spots”, thereby achieving high sensitivity and quantitative detection. This strategy was successfully applied to detect trace antibiotic nitrofurazone on the surface of aquaculture shrimp, showing good linearity in the concentration range from 10^−4^ M to 10^−10^ M, with a LOD as low as 10^−9^ M (approximately 0.02 μg/kg), which surpasses relevant European Union standards.

To meet point-of-care testing needs, the combination of LFIA with SERS has become a research hotspot. Tian et al. [32] developed an LFIA based on SERS. In this study, 1,4-benzenedithiol (BDT) was selected as the Raman reporter molecule. BDT-mediated gap gold nanorod@gold core–shell nanoparticles (AuNR@Au NPs) were synthesized and used as the SERS substrate to construct immunoprobes for the detection of enrofloxacin in food samples. The entire assay can be completed within 15 min. Quantitative analysis based on SERS signals showed that the half-maximal inhibitory concentration (IC50) and LOD for enrofloxacin were 59 pg mL^−1^ and 0.12 pg mL^−1^, respectively (equivalent to 71 pg g^−1^ and 0.14 pg g^−1^ in real samples). The recoveries from spiked samples (shrimp meat, swine meat, and swine liver) ranged from 89.2% to 102.4% with relative standard deviations (RSD) of 1.70–6.38%. Pan et al. [28] employed 4-mercaptobenzoic acid (4-MBA)-labeled Au@Ag core–shell nanoparticles as SERS probes to develop a SERS-LFIA for detecting chloramphenicol antibiotics in aquatic products. The LODs for chloramphenicol, thiamphenicol, and florfenicol reached 0.36, 0.20, and 0.78 ng/mL, respectively. Min et al. [33] established a method combining thin-layer chromatography (TLC) with SERS for the rapid detection of quinolone antibiotic residues in aquatic products. The study used ciprofloxacin, norfloxacin, lomefloxacin, and levofloxacin as the research subjects, employing silver sol as the SERS-active substrate. Under optimized chromatographic conditions, the separation and detection of the four antibiotics were achieved.

In summary, current trends in SERS technology for antibiotic detection are characterized by: (1) Focus on functional integration of substrates, combining magnetic separation, membrane-based enrichment, and SERS-active units to simplify pretreatment and improve selectivity; (2) Advancement towards portable, point-of-care detection modes, with SERS-LFIA being a successful example; (3) Extensive exploration of novel materials like MXeneto synergize electromagnetic and chemical enhancement, thereby improving sensitivity and specificity. Future challenges may lie in further reducing substrate cost, improving batch-to-batch reproducibility, and developing more universal sample pretreatment methods to promote the transition of SERS technology from the laboratory to broader on-site regulatory applications.

SERS technology’s main advantages lie in its ability to provide molecular structural information with a “fingerprint” identification characteristic and its extremely high, potentially single-molecule-level sensitivity, showing great potential for identifying specific antibiotics in complex mixtures. However, its signal intensity heavily relies on the uniformity and stability of the nanostructured enhancement substrates, leading to reproducibility issues, and the preparation cost for high-performance substrates is relatively high. Compared to simple-to-operate CRS or LFIA, SERS typically requires a professional spectrometer and a trained operator, posing major obstacles to its widespread application in terms of portability, total analysis cost, and operational complexity.

### 4.4. ELISA

**Figure 7:**
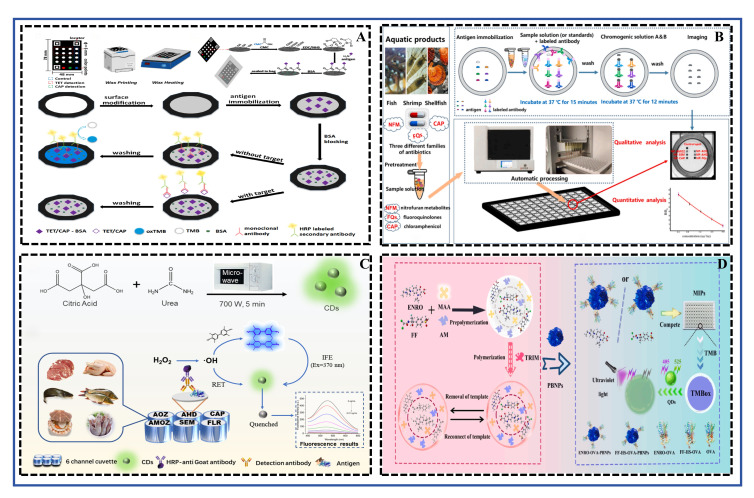
Working principle of ELISA for detecting antibiotic residues in aquatic products. (**A**): Schematic diagram illustrating the principle of highly sensitive ELISA−based detection of TC and CAP in aquatic products using PAS [21]. (**B**): Workflow and schematic diagram of protein microarray biochip detection of multiantibiotic residues in aquatic products [22]. (**C**): Preparation method of the CDs fluorescent probes and test procedures of FQIAs [25]. (**D**): Schematic illustration of fluorescence quenching BELISA based on QDs [26].

ELISA is a highly sensitive detection technique that combines specific antigen–antibody binding reactions with enzyme-catalyzed chromogenic reactions. Its core principle involves immobilizing an antigen or antibody on a solid phase and using an enzyme conjugate to catalyze a substrate, producing a detectable signal (e.g., color, fluorescence) for the qualitative or quantitative analysis of the target. In food safety detection, particularly for antibiotic residues, the indirect competitive ELISA is one of the most commonly used formats. In this format, free target in the sample competes with the antigen immobilized on the solid phase for binding to a limited amount of specific antibody. Subsequently, an enzyme-labeled secondary antibody and substrate are used for color development, where the signal intensity is inversely proportional to the target concentration in the sample [20,21]. This principle forms the foundation for numerous methodological innovations. The schematic diagram illustrating the principles of both single and multiplex detection of antibiotic residues in aquatic products using ELISA was shown in Figure 7.

The widespread application of ELISA is primarily due to its significant advantages: high sensitivity and specificity, relatively simple operation, suitability for high-throughput screening, and low cost. Compared to confirmatory methods like LC-MS, ELISA does not require expensive instruments or complex sample preparation, making it more suitable for on-site rapid screening and preliminary testing of large sample batches. Researchers are continuously strengthening these advantages through material innovation and detection mode optimization. For instance, developing paper-based sensors aims to reduce costs and enhance on-site detection capability [21]; while designing broad-spectrum antibodies aims to achieve simultaneous multi-residue detection, improving screening efficiency [20].

Wang et al. [21] developed a paper-based antibiotic sensor fabricated simply by wax printing and surface modification, with a cost as low as approximately $0.15 per test. This method ingeniously transferred the traditional microplate ELISA to a paper platform and combined it with smartphone image analysis, achieving on-site quantitative detection of tetracycline and chloramphenicol. This marks an important step in the transition of ELISA technology from the laboratory towards portable, user-friendly on-site detection. Zhang et al. [20] focused on improving detection throughput. They utilized a monoclonal antibody with broad-spectrum recognition capability for ofloxacin to establish an ic-ELISA that could simultaneously detect 13 fluoroquinolone antibiotics. This strategy of “one antibody detecting a class of drugs” effectively addresses the challenges of limited antibody libraries and high costs in multi-residue screening, providing an efficient solution for monitoring structurally similar contaminants. Further integration and signal amplification represent another important direction. Tang et al. [22] first reported a visual protein microarray chip based on a 96-well plate, integrating nano-silver catalytic color development and fully automated plate reading. It could high-throughput screen nitrofuran metabolites, chloramphenicol, and fluoroquinolones in aquatic products within 60 min. This work combines the high-throughput nature of ELISA with the multiplexing advantages of microarrays and the catalytic amplification effect of nanozymes, representing an important trend towards integrated, automated, multi-target rapid detection platforms. Additionally, other studies have introduced novel signal probes to enhance sensitivity. For example, Hu et al. [26] utilized fluorescence quenching biomimetic ELISA and a nanozyme catalytic strategy to detect enrofloxacin and florfenicol; Li et al. [25] employed a hydroxyl radical-enhanced carbon dot fluorescence quenching immunoassay to achieve highly sensitive detection of six antibiotics. These studies, by fusing nanomaterials and novel catalytic mechanisms, significantly enhanced the signal output of traditional ELISA, continually pushing for lower detection limits.

In terms of performance, these methods demonstrate excellent sensitivity, specificity, and accuracy. The paper-based sensor by Wang et al. [21] achieved LODs of 0.5 ng/mL for tetracycline and 0.05 ng/mL for chloramphenicol. Compared with HPLC results in real milk and fish samples, the accuracy was no less than 97.9%. The broad-spectrum ic-ELISA by Zhang et al. [20] showed an IC_50_ of 19.23 μg/L for enrofloxacin, with cross-reactivity rates above 85% for the other 12 fluoroquinolones and below 0.2% for antibiotics of other classes, indicating high intra-class specificity and good inter-class selectivity. In spiked recovery experiments with real bullfrog samples, the results were highly consistent with HPLC (R^2^ = 0.9936). The visual chip by Tang et al. [22] achieved LODs ranging from 0.07 to 6.42 μg/kg for the three antibiotic classes, with average recoveries of 89.4–111.2%, meeting quantitative detection requirements. The study by Khan and Livel [24] validated the reliability of commercial ELISA kits from an application perspective. Their use for screening multiple antimicrobial residues in imported shrimp yielded results consistent with LC-MS/MS confirmation, affirming the important value of ELISA as a reliable preliminary screening tool in actual regulatory work. In summary, the methods in the literature not only enhance performance through innovative design but also prove their accuracy and practicality in real sample detection, continuously advancing the development of ELISA technology in the field of food safety monitoring.

ELISA, as a classic immunoassay method, offers high specificity, good reproducibility, and the advantage of processing a large number of samples simultaneously, making it suitable for batch screening in laboratories and small on-site labs. However, its analytical procedure involves multiple steps, is time-consuming (typically 1–3 h), and requires specialized equipment such as a microplate reader, which limits its application in extreme on-site conditions. Compared to rapid, disposable sensors (e.g., ECRS), ELISA does not have an advantage in detection speed and device integration; and compared to LFIA, its operation is considerably more complex.

### 4.5. ECRS

**Figure 8:**
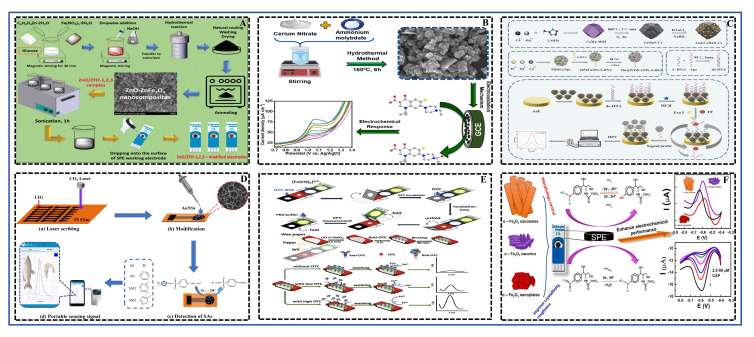
Working principle of electrochemical sensor for detecting antibiotic residues in aquatic products. (**A**): Schematic illustration of the synthesis route for ZnO/ZFO nanocomposites and the fabrication process of ZnO/ZFO modified SPE for the electrochemical detection of furazolidone (FZD) [36]. (**B**): Schematic representation of the synthesis of cerium molybdate and its sensing application [38]. (**C**): Schematic diagram of synthesis of Au@CoMnN−Cs, Thi@PtPdCu NPs−S−DNA, ds−DNA and aptasensor detection of FF [39]. (**D**): Fabrication of AuNS/LIPG electrodes and SA detection [40]. (**E**): Preparation and working operation of the OTC biosensor [42]. (**F**): Schematic illustration of the enhanced mechanism of electrochemical sensing performance for CAP using SPE electrode modified with α−Fe2O3−T, α−Fe2O3−R and α−Fe2O3−P [43].

ECRS achieve quantitative detection by measuring changes in current, potential, or impedance generated during the redox reaction of the target analyte on the electrode surface. The core lies in converting chemical signals into measurable electrical signals [37]. In recent years, they have shown great application potential in food safety monitoring, especially for antibiotic residue detection, primarily due to their methodological advantages: relatively simple instrumentation, convenient operation, rapid response, low cost, and ease of miniaturization and on-site rapid detection. The schematic diagram illustrating the principle of detecting antibiotic residues in aquatic products using ECRS was shown in Figure 8.

To improve detection sensitivity and selectivity, research focuses on developing novel nanomaterials and introducing specific biological recognition elements. In direct detection strategies, high-performance nanomaterials are employed as electrode modifiers to enhance electron transfer kinetics and electrocatalytic activity toward the target. For example, Anh et al. [36] constructed a sensor using a ZnO/ZnFe_2_O_4_ heterojunction nanocomposite to detect furazolidone. The formation of the heterojunction effectively promoted electron transport, resulting in a good linear range of 1–100 μM and an LOD of 0.65 μM. Similarly, Huyen et al. [43] optimized the electrochemical performance by tuning the morphology of α-Fe_2_O_3_ nanostructures for chloramphenicol detection. Pervaiz et al. [38] employed hydrothermally synthesized cerium molybdate (Ce_2_(MoO_4_)_3_) nanomaterials, leveraging their excellent electrocatalytic performance to achieve highly sensitive detection of enrofloxacin with an LOD of 0.035 μM. These works demonstrate that well-designed nanomaterials are key to enhancing sensor analytical performance. Notably, the work by Qiu et al. [40] combined material innovation with device portability. They developed a flexible electrode based on gold nanoshell-modified laser-induced porous graphene (AuNSs/LIPG) and integrated it with a wireless portable device, enabling rapid screening of sulfonamides within 6 s. This marks a solid step towards point-of-care testing for ECRS.

Alternatively, sensing strategies incorporating biological recognition elements, particularly aptamer-based sensors, significantly enhance detection selectivity. Such sensors often employ a “signal-switching” mode. For example, Jampasa et al. [42] constructed a label-free paper-based electrochemical immunosensor for detecting oxytetracycline via a competitive format. Its “signal-on” mechanism stemmed from the change in electrode interface properties after oxytetracycline competed with the immobilized antigen for antibody binding, increasing the accessibility of the electrochemical probe [Fe(CN)_6_]^3−^/^4−^. More studies use a “signal-off” mode, such as the aptasensor developed by Wang et al. [39] for detecting florfenicol. It employed a dual strategy of target-triggered enzyme cycling amplification and nanomaterial signal amplification, achieving ultra-high sensitivity (LOD = 5.28 × 10^−4^ ng mL^−1^). The work by Xu et al. [37] demonstrates ingenious integrated design. The synthesized Ce-MOF-808@CeO_2_ nanocomposite possessed dual functions: nanozyme activity and an aptamer immobilization platform. When tetracycline bound to the aptamer, the resulting steric hindrance inhibited the catalytic reduction current of H_2_O_2_ by the composite, enabling ultra-sensitive detection of tetracycline (LOD = 0.21 pM). Que et al. [41] also utilized the synergistic effect of polyoxometalates and organic ligands to construct a sensing interface for detecting sulfathiazole.

In summary, current trends in electrochemical sensor development show two clear directions: first, striving to continuously push the limits of detection sensitivity through nanoengineering and signal amplification techniques like hybridization chain reaction and enzyme cycling; second, focusing on practical application by developing flexible electrodes, portable readout devices, and integration with smartphones to advance sensors from the laboratory to the field. However, how to further ensure sensor stability, reproducibility, and anti-interference capability in complex real sample matrices, as well as how to realize integrated sensor arrays for simultaneous detection of multiple contaminants, remain key challenges for future research.

ECRS have attracted significant attention due to their high sensitivity, rapid response, ease of miniaturization, and relatively low-cost potential. They are also well-suited for integration with portable readers or smartphones to achieve quantitative detection. The core challenges to their performance lie in the anti-fouling capability and long-term stability of the electrode interface, as they are susceptible to interference in complex biological matrices and often exhibit poor reusability. Compared to SERS, they offer lower equipment cost and complexity; however, compared to LFIA or intuitive CRS, they still require basic electrical readout devices, which slightly compromises user-friendliness. In terms of achieving multiplex detection, feasibility exists through the design of electrode arrays.

### 4.6. CRS

**Figure 9:**
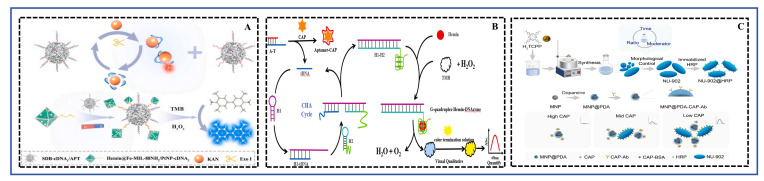
Working principle of CRS for detecting antibiotic residues in aquatic products. (**A**): Schematic illustration of the colorimetric aptasensor for KAN detection based on a "three−in−one" synergistic nanozymes assisted with Exo I amplification [14]. (**B**): Schematic of a colorimetric aptasensor based on CHA and G−quadruplex for CAP detection [16]. (**C**): Demonstration of NH signaling probe synthesis and the mediated immunosensor [17].

As an important rapid detection technology, the core principle of CRS lies in converting the specific recognition event between the target analyte and the sensing element into a visible color change or a measurable absorbance change. This conversion is typically achieved using nanomaterials with catalytic activity (e.g., nanozymes) or functional nucleic acids (e.g., aptamers). For instance, some sensors utilize nanozymes with peroxidase-like activity to catalyze chromogenic substrates (e.g., 3,3′,5,5′-tetramethylbenzidine, TMB) to produce a color change [14]. Others rely on conformational changes in aptamers upon target binding, which modulate the activity of G-quadruplex/hemin DNAzymes [16] or alter the aggregation state of dyes (e.g., cyanine 7, Cy [15]), thereby changing the system’s absorbance. The prominent advantages of this method are its simple operation, rapid response, low cost, and the ability to achieve visual semi-quantitative judgment without complex instruments, making it particularly suitable for on-site rapid screening and high-throughput detection. The schematic diagram illustrating the principle of detecting antibiotic residues in aquatic products using a CRS was shown in Figure 9.

In the analyzed literature, researchers designed distinctive signal amplification and transduction strategies for different antibiotic targets, significantly improving sensor performance. For example, Ji et al. [17] synthesized a monodisperse nanoscale MOF NU-902 with uniform particle size and immobilized horseradish peroxidase (HRP) on its surface, constructing a highly catalytically active colorimetric signal probe named NU-902@HRP (NH). Concurrently, the study employed polydopamine-coated magnetic nanoparticles (MNP@PDA) as immunoreaction carriers, enabling rapid separation under an external magnetic field and reducing antibody consumption by 80%. This NH biosensor, based on a direct HRP-TMB colorimetric system, achieved the detection of chloramphenicol within 38 min. It demonstrated a linear range of 0.05–1000 ng mL^−1^ and a LOD as low as 0.0184 ng mL^−1^ (approximately 0.057 nM), exhibiting excellent performance in the analysis of various real samples such as shrimp, eggs, and milk. The work by Li et al. [14] focused on improving the detection sensitivity for kanamycin (KANA). They constructed a “three-in-one” nanohybrid integrating iron oxide, gold nanoparticles, and platinum nanoparticles, which exhibited excellent synergistic nanozyme activity. Combined with the target recycling amplification effect of exonuclease I (Exo I), this aptasensor achieved an ultra-low LOD of 2 pg mL^−1^. This research demonstrated that carefully designing nanomaterials to optimize their intrinsic catalytic activity, combined with enzyme-assisted target recycling, was a powerful means to achieve ultra-sensitive detection.

To address the detection needs in real samples where multiple analogues may coexist, developing broad-spectrum sensing strategies is particularly important. Xu et al. [15] adopted an innovative “multi-target” SELEX technology to successfully screen a broad-spectrum aptamer capable of simultaneously and specifically binding to five different sulfonamide antibiotics. Based on this aptamer and a Cy7 dye displacement strategy, they developed a CRS that could simultaneously screen for the five SAs directly through a solution color change from colorless to blue, with visual detection limits of 0.2–0.5 μM. This work broke through the limitation of traditional aptasensors typically detecting only a single target, providing a highly practical solution for the total screening of multiple antibiotic residues in animal-derived foods. On the other hand, Wang et al. [16] designed a dual-amplification aptasensor based on catalytic hairpin assembly (CHA) and G-quadruplex formation for detecting chloramphenicol in aquatic products. This strategy utilized the CHA reaction to achieve target recycling while simultaneously generating numerous G-quadruplexes with peroxidase-like activity, thereby amplifying the colorimetric signal and ultimately achieving excellent sensitivity with an LOD of 90 pg/mL. This exemplifies the great potential of enzyme-free isothermal amplification techniques and functional nucleic acid self-assembly in constructing highly sensitive biosensors.

Overall, the performance of these CRS is outstanding, with detection limits covering the range from pM to μM, and they also perform well in terms of linear range, selectivity, and recovery rates (typically between 80 and 120%) in real samples (e.g., fish, honey, milk). These research advances highlight the application value of CRS in food safety monitoring. However, it should be noted that further improving specificity in complex matrices, simplifying pretreatment steps, and promoting the development of sensors towards portable, integrated devices remain directions that require continuous attention and breakthroughs in this field.

CRS offer the greatest advantage in that their detection results can be semi-quantitatively interpreted directly by the naked eye through observable color changes, or quantitatively via smartphone cameras. This provides an intuitive interface and requires minimal equipment, making them highly suitable for grassroots and consumer applications. Their performance largely depends on the stability of the nanoprobes (e.g., gold nanoparticles) and the interference resistance of the colorimetric reaction. While their sensitivity is generally lower than that of fluorescence or electrochemical methods, it is sufficient to meet the screening requirements for many residue limits. Compared to LFIA, solution-phase colorimetric assays offer greater flexibility for automation and high-throughput analysis, though they may involve slightly more operational steps. Their cost per test is highly competitive, and they exhibit good scalability.

### 4.7. Key Trends in Existing Methods

As can be seen from the detection limit column in Table 1, while SERS and ECRS may achieve extremely low LODs under ideal conditions, in complex real sample matrices (such as whole shrimp or fish homogenate), LFIA and CRS may demonstrate more stable and reliable detection limits due to their simpler pretreatment procedures and designs that resist matrix interference. From the precision column in Table 1, it is evident that ELISA and FRS/ECRS based on mature readers generally offer better reproducibility. In contrast, the interpretation of results for LFIA and CRS may rely more on subjective visual judgment or smartphone cameras, potentially leading to slightly greater variation between batches or operators. Regarding the universality of materials and technologies, gold nanoparticles have been widely and successfully used in both LFIA and CRS for detecting different antibiotics, indicating their stability and versatility as signal labels. Quantum dots and MOF materials are frequently employed in numerous FRS, benefiting from their high fluorescence quantum yield and adsorption properties. Certain aptamers or molecularly imprinted polymers have also been successfully applied to detect multiple classes of antibiotics. In terms of future translational potential, LFIA test strips are currently the most mature in commercial conversion and on-site application due to their extreme simplicity, speed, low cost, and portability. Smartphone-integrated CRS/FRS hold significant translational promise because they do not require specialized equipment and have a broad user base. In contrast, SERS and some high-precision ECRS, possibly limited by cost, operational complexity, or device portability, are currently more at the laboratory validation stage.

The preceding separate elaboration of the six technologies has elucidated their respective technological pathways and distinct strengths. However, to identify their appropriate niches in practical applications, a side-by-side comparison is essential. The following discussion will compare them side-by-side across multiple dimensions, including sensitivity, resistance to interference, multiplex detection capability, operational complexity, difficulty of on-site deployment, cost, and stability, and will outline the common challenges they face.

## 5. Challenges and Solutions in Existing Detection Methods

Currently, the primary challenge for on-site rapid detection of antibiotic residues in aquatic products is the severe interference from complex sample matrices. Components such as proteins, fats, amino acids, and sugars in aquatic products can non-specifically adsorb onto sensor surfaces or mask target signals, significantly reducing detection accuracy and sensitivity [136]. To address this, researchers have developed efficient sample pretreatment and signal separation strategies. For example, the development of magnetic SERS substrates, such as Ti_3_C_2_T_x_/Fe_3_O_4_/Ag, enables the integration of magnetic separation, enrichment, and matrix purification of targets, effectively avoiding cross-contamination and improving method selectivity [30]. In the field of immunochromatography, optimized universal pretreatment methods have been reported. Using ethyl acetate extraction combined with a derivatization step allows for the simultaneous preparation of samples for four classes of banned substances (nitrofuran metabolites, chloramphenicol, and malachite green) within 40 min, greatly enhancing the efficiency of multi-residue synchronous detection [54]. This study posited that future research should focus on developing more universal and rapid “one-step” or “extraction-free” pretreatment technologies, particularly modules integrated with portable devices, which are crucial for achieving true point-of-care testing.

Insufficient detection sensitivity is another core bottleneck restricting the accurate on-site detection of antibiotic residues in aquatic products, especially when needing to meet increasingly stringent MRLs. The visual detection limits of traditional CRS or colloidal gold immunochromatographic test strips sometimes struggle to meet regulatory requirements [15,47]. Consequently, the field seeks breakthroughs through signal amplification and conversion strategies. On one hand, novel functional nanomaterials are designed and applied to enhance signals. For instance, using metal-polydopamine frameworks or hyperbranched gold plasmonic blackbodies as labels, whose signal intensity far exceeds that of traditional gold nanoparticles, can improve the detection limit of immunochromatography by tens of times [51]. On the other hand, developing ratiometric or dual-mode signal output sensors reduces environmental interference through self-calibration mechanisms, improving quantitative reliability. For example, a colorimetric/fluorescent dual-signal immunochromatographic method based on PCN-222 achieved a sensitivity ten times greater than that of the single colorimetric mode [46,53]. Future directions for enhancing sensitivity include the development of intelligent, responsive nanomaterials with superior catalytic or plasmonic properties and the exploration of novel sensing mechanisms enabling cascade amplification, such as the effective integration of isothermal nucleic acid amplification techniques with portable readout platforms.

Faced with the realistic scenario of potential co-residues of multiple antibiotic classes in aquatic products, achieving high-throughput, multi-target synchronous detection is a significant methodological challenge. Traditional single-target detection methods are inefficient and cannot comprehensively reflect the contamination status of samples. Existing solutions mainly revolve around constructing multiplex detection platforms. For instance, employing multiple test lines to label distinct antibodies, a study successfully developed a multiplex immunochromatographic strip capable of simultaneously detecting 83 antibiotics across five major classes [48]. In the field of fluorescence sensing, combining aptamers recognizing different antibiotics with metal–organic frameworks or carbon dots exhibiting specific fluorescent responses enables the ratiometric or spatially resolved synchronous detection of multiple antibiotics [60,64]. This study contended that future multi-target detection technologies should develop towards higher integration and greater intelligence. For example, developing array-based sensors on microfluidic chips, combined with machine learning algorithms for pattern recognition of multi-channel signals, could not only identify types but also simultaneously quantify concentrations, which would be a powerful tool for addressing complex residue scenarios.

Achieving rapid, convenient on-site detection and even real-time monitoring is the ultimate goal of food safety supervision. However, this requires detection methods to overcome reliance on large instruments and professional operators. Many current high-sensitivity methods, such as HPLC-MS, though accurate, are difficult to deploy on-site. Solutions in the literature focus on developing portable devices and simple readout systems. A prominent trend is the integration of sensors with smartphones, using the phone’s camera to capture color or fluorescence changes and dedicated applications for quantitative analysis [58,61,68]. Additionally, paper-based microfluidic chips combined with colorimetric or fluorescent detection, with their advantages of low cost, portability, and disposability, provide powerful tools for on-site screening [53,60]. This study predicted that future on-site detection technologies will integrate more deeply with the Internet of Things and cloud computing. For instance, developing intelligent sensing patches or test strips capable of wirelessly transmitting data directly to cloud-based regulatory platforms could enable real-time monitoring and risk warning throughout the entire chain from production to circulation.

Furthermore, method universality, stability, and cost control are non-negligible challenges in practical application. Sensing strategies developed for a specific antibiotic are often not directly applicable to other structural analogues, while the synthesis and modification of complex nanomaterials may increase costs and cause batch-to-batch variations. Existing studies address the universality challenge by screening broad-spectrum recognition elements, such as developing antibodies or aptamers that can simultaneously recognize multiple sulfonamides or fluoroquinolones [15,20,48]. To reduce costs, research strives to use cheaper, more readily available materials, such as laser-induced graphene flexible electrodes, or to optimize synthesis processes for preparing nanomaterials with stable performance and high reproducibility [29,40]. The insight of this study was that future research should pay more attention to an “application-oriented” design philosophy. Factors such as detection performance, cost, manufacturability, and environmental friendliness should be comprehensively considered from the early stages of development. Exploring the use of biodegradable or sustainably synthesized nanomaterials as sensing elements and promoting the modular, standardized production of detection devices are significant for advancing these detection technologies from the laboratory to the market, truly serving daily regulatory needs.

## 6. Scalability and Practical Deployment Challenges

While various on-site rapid detection technologies have demonstrated excellent performance in laboratory research, their transition from laboratory proof-of-concept to scaled-up production and practical field deployment still faces a series of common industrialization and practical challenges. An in-depth discussion of these challenges is crucial for the genuine realization of these technologies.

The excellent performance achieved at the laboratory scale is often difficult to replicate in batch production. For technologies reliant on nanomaterials (e.g., gold nanoparticles, MOFs for signal amplification) or biological recognition elements (e.g., monoclonal antibodies, aptamers), ensuring high batch-to-batch consistency during large-scale synthesis is a significant challenge. For instance, the performance of LFIA strips is highly dependent on the uniformity of antibody line deposition on the nitrocellulose membrane and the stability of gold-labeled antibodies; minor fluctuations in these process parameters can affect detection sensitivity and reproducibility. Similarly, the performance of electrochemical or SERS sensors is intimately linked to the nanostructure of the electrode or substrate, making reproducible and low-cost micro/nano-fabrication techniques key to scalable manufacturing.

Cost is a core factor determining whether a technology can be widely adopted and must be considered from two perspectives: initial device investment and cost per test. CRS and LFIA strips typically require no expensive equipment and have an extremely low cost per test, giving them a significant cost advantage. In contrast, smartphone-based fluorescence or electrochemical sensing platforms, while leveraging the user’s smartphone, still incur costs for the necessary portable optical attachments or miniaturized potentiostats. SERS technology and some high-precision electrochemical workstations involve higher equipment investments. Furthermore, the reagents, consumables, and time required for complex sample pretreatment should also be factored into the total analysis cost. An ideal on-site detection technology should seek the optimal balance between device cost and consumable cost while meeting performance requirements.

On-site detection tools often need to be stored and transported under non-laboratory conditions, making their storage stability (shelf life) paramount. This is primarily constrained by the activity decay of biological recognition elements (antibodies, enzymes, nucleic acid aptamers) and signal probes (e.g., enzyme conjugates, fluorescent dyes) in either dried or liquid forms. For example, LFIA strips need to maintain long-term stability at ambient temperature, placing extremely high demands on antibody and label immobilization technologies. Developing effective lyophilization processes, stabilizer formulations, and robust reagent storage compartments are important research directions for extending device shelf life and ensuring reliable detection results.

Any detection method must undergo a rigorous validation process to gain acceptance from regulatory agencies (e.g., the U.S. FDA, the EU EFSA, China’s Ministry of Agriculture and Rural Affairs) for use in official monitoring. This involves systematic evaluation of its sensitivity, specificity, accuracy (recovery), precision, robustness, etc., along with comparative validation against current national standard methods (e.g., HPLC and HPLC-MS/MS), as seen with commercially mature technologies like LFIA [45,47,49,50] and ELIS [20,21,22,26]. A fundamental requirement is that the detection limit of the rapid on-site method must be below, or significantly below, the Maximum Residue Limits (MRLs) for the relevant antibiotics in specific aquatic products. Integrating these methods into existing monitoring frameworks as pre-screening tools prior to confirmatory mass spectrometry analysis would be highly beneficial. Currently, most emerging sensing technologies remain in the academic research stage and lack large-scale inter-laboratory collaborative validation data, which is a major obstacle to their regulatory acceptance. Establishing validation protocols equivalent or correlated to national standard methods is a necessary step for technology transfer.

In summary, technology transfer is a systematic engineering project involving the entire chain of “R&D-pilot-scale-mass production-regulation-market”. Beyond the specific challenges mentioned above, it also faces non-technical barriers such as competition from traditional laboratory testing methods, cultivation of user habits, establishment of quality assurance and control systems, and clear market demand and business models. Deep integration between academia and industry, targeted product development for specific application scenarios (e.g., aquaculture farms, ports, markets), and early consideration of cost, stability, and regulatory requirements in the R&D phase are key to accelerating the commercialization of these promising on-site detection technologies.

Understanding the aforementioned challenges and their emerging solutions ultimately serves the purpose of making prudent selections and deploying technologies wisely. There is no one-size-fits-all technology; the optimal choice depends heavily on the specific application scenario, available resources, and performance requirements. In the following section, building on the comparative analysis presented earlier, we will recommend the most suitable technology portfolios for different segments of the industry chain.

## 7. On-Site Detection Method Selection Based on Different Application Scenarios

The optimal choice of an on-site detection technology is not universal but heavily depends on the specific requirements, constraints, and operational context of the application scenario. To guide this selection, Table 4 provides a high-level overview of the suitability and, crucially, the current commercialization readiness of the six reviewed technologies across three major field implementation scenarios.

The following subsections elaborate on the rationale behind these recommendations, with explicit reference to the commercialization stages outlined in Table 4.

### 7.1. Farm-Side Real-Time Monitoring

At the aquaculture site, the core challenge lies in performing large-scale, high-throughput preliminary screening of numerous samples for multiple antibiotic classes rapidly and cost-effectively, a demand traditional lab methods struggle to meet. Here, the technological development focus is on enhancing “multiplex detection” capability alongside standardized pretreatment, rather than pursuing ultimate single-analyte sensitivity. For example, multiplex immunochromatographic test strips based on gold nanoparticles have been developed to simultaneously detect dozens of antibiotics, providing a powerful tool for source monitoring [48].

The commercialization readiness of technologies for this scenario varies significantly, as outlined in Table 4. LFIA test strips, especially in multiplex formats, are the mature and preferred solution that directly addresses the need for simplicity, speed, and multi-target screening, with established commercial products available. For applications requiring semi-quantitative data or digital record-keeping, smartphone-based colorimetric platforms and simpler ECRS are the most promising emerging, pre-commercial technologies under active field validation. In contrast, while offering high sensitivity, SERS and sophisticated fluorescent platforms remain primarily in the academic research domain for on-farm use due to barriers of cost, operational complexity, and a current focus on single- or limited-plex detection in most research prototypes.

### 7.2. Rapid Screening in Processing and Distribution Channels

This scenario encompasses on-site spot checks in settings like farmers’ markets and supermarkets, as well as rapid inspections by authorities such as customs and market supervision agencies. The core challenge is to enable non-professionals to perform accurate quantitative or reliable semi-quantitative analysis of specific, high-risk antibiotic targets within a short timeframe (typically 10–30 min) in non-laboratory environments. Therefore, the ideal methods for this scenario must successfully combine good portability, simple and intuitive operation, and acceptable quantitative reliability.

As outlined in Table 4, technologies with established protocols and better precision are particularly valued here. Portable ELISA readers and dedicated electrochemical analyzers have a commercialized presence in this segment, offering validated performance suitable for regulatory compliance. LFIA strips serve as an indispensable tool for rapid, low-cost pre-screening.

A prime example of the trend towards integrated, user-friendly quantitative platforms is the development of sensing systems that couple specific recognition elements (e.g., aptamers, antibodies) with portable signal readout devices, especially smartphones. For instance, a multicolor fluorescence sensing strategy based on aptamers and carbon dots was integrated into a paper-based microfluidic chip. This chip, combined with a custom 3D-printed detection box and a smartphone for color recognition, enabled the visual and semi-quantitative detection of three antibiotics (sulfamethazine, oxytetracycline, and chloramphenicol) in shrimp simultaneously within 1 min [60]. Similarly, fabricating fluorescent metal–organic framework materials into agarose gel test strips allows for smartphone-based, on-site visual quantitative analysis of contaminants like malachite green [8]. The core advantage of such integrated platforms lies in condensing the analytical process into a simple workflow: “sample loading → photographing → result reading”, which significantly lowers the technical barrier for operators while ensuring data objectivity and traceability. These smartphone-based platforms (colorimetric/fluorescent) are currently in the pre-commercial or advanced prototyping stage (see Table 4) and represent the most promising direction for bridging the gap between laboratory-grade quantification and field-deployable rapid screening.

In contrast, while SERS technology demonstrates outstanding discriminatory power and sensitivity in laboratory research, it currently remains primarily in the domain of academic research and high-end customized solutions for this application. The barriers include the high cost of robust, field-portable spectrometers and the lack of standardized, simple protocols, limiting its widespread near-term adoption in routine screening channels.

### 7.3. Consumer-End Self-Testing and Market Surveillance

For end-user applications at the consumer level (e.g., households, restaurants), the challenges are paramount: users typically possess minimal technical expertise and exhibit high sensitivity to cost. Detection methods must, therefore, prioritize extreme simplicity, intuitive result interpretation, and low cost. The suitability and commercialization status of technologies for this demanding scenario are summarized in Table 4.

Mature and Dominant Technology: In this context, LFIA test strips, which require no instrumentation and rely on visual readout, are the most commercially mature and widely available solution, as indicated by their “High” readiness in Table 4. Current research and development focus on enhancing their performance within this familiar format. Efforts are directed at improving sensitivity and reliability through advanced signal labels to offset inherent limitations in quantitative precision. For instance, substituting traditional colloidal gold with novel nanomaterials like metal-polydanamine frameworks or rare-earth-doped nanoparticles can significantly amplify colorimetric or fluorescent signals, thereby lowering the visual detection limit [45,49]. Furthermore, the development of multiplex test strips capable of displaying results for several targets simultaneously, though currently limited (e.g., a triplex strip for neomycin, penicillin, and chloramphenicol [44]), represents a significant step towards providing consumers with a more comprehensive safety profile.

Emerging and Pre-commercial Technology: As shown in Table 4, smartphone-based colorimetric detection is the primary technology in the pre-commercial validation stage with high potential for this market. It aligns perfectly with the trend of leveraging ubiquitous devices to enhance accessibility and data record-keeping. The overarching design principle for consumer-facing technologies prioritizes “ease of use” and “instant result clarity” (e.g., an unambiguous line or color change) over sophisticated multiplexing capabilities. Consequently, other technologies that necessitate the purchase of a dedicated portable reader (e.g., for fluorescence or electrochemistry) face a significant adoption barrier for the general public and are more likely to be commercialized for professional or institutional users.

In summary, selecting an on-site rapid detection technology is not a one-size-fits-all process, but rather a decision that requires balancing detection requirements, scenario constraints, and technology readiness. The scenario-based selection framework presented in this chapter, combined with a clear assessment of the commercialization readiness of each technology, aims to provide a practical decision-making tool for various stakeholders across the supply chain, from aquaculture farms to end consumers. With ongoing advances in materials science, microfluidics, and artificial intelligence, future on-site detection platforms will evolve to be more integrated, intelligent, and accessible. The evaluation dimensions and framework proposed here will also dynamically adapt, continuing to provide a reference for technology selection in safeguarding aquatic product safety.

## 8. Conclusions

### 8.1. Main Conclusions

This review systematically organizes and compares six mainstream on-site rapid detection technologies for antibiotic residues in aquatic products applied between 2021 and 2025. Analysis indicates that the field is advancing towards higher sensitivity, stronger specificity, more convenient operation, and intelligent readouts. FRS and ECRS generally exhibit excellent sensitivity and quantitative precision, but they often require dedicated equipment. LFIA and CRS (especially those on smartphone platforms) excel in operational simplicity, cost, and portability, making them most suitable for grassroots and consumer-end applications. SERS provides unique “fingerprint” identification capabilities, but equipment cost and signal reproducibility remain bottlenecks for its on-site application. The ELISA, as a mature method, maintains an important role in the batch screening of samples. The choice of technology is highly dependent on the specific application scenario: in resource-limited environments like farms, techniques requiring no instruments, such as lateral flow strips, should be prioritized; at regulatory points like ports, portable ECRS or FRS with higher sensitivity are preferable; and for consumer self-testing, smartphone-integrated CRS show the greatest potential. Successful future technological solutions will inevitably represent a comprehensive balance of detection performance, cost, ease of use, and integration with existing workflows. To provide an intuitive comparison, Table 5 presents a qualitative summary and rating of the aforementioned technologies across six key dimensions, aiming to offer a quick reference for technology selection in different application scenarios.

### 8.2. Limitations of This Review

This study has the following limitations: First, the review focuses on six mainstream technologies, FRS, LFIA, SERS, ELISA, ECRS, and CRS, potentially not fully covering other emerging sensing principles that are still in early laboratory stages but show considerable promise. Second, most of the reviewed studies validate performance under controlled laboratory conditions; data on their large-scale, long-term stability and reliability in real, complex, and dynamic aquaculture, processing, and distribution environments remain relatively scarce, limiting an accurate assessment of the practical robustness of these technologies. Finally, as most original studies do not provide detailed commercialization cost analyses, the cost comparison of different technologies in this paper relies more on inferences from components and processes rather than actual market data, which imposes certain limitations on precise quantification.

### 8.3. Future Perspectives and Research Priorities

To advance the field from the laboratory to practical on-site application, future studies should focus on the following priorities: (1) Probe and Material Innovation: Develop novel recognition elements (e.g., high-affinity aptamers, molecularly imprinted polymers) that are more stable, inexpensive, and possess broad-spectrum recognition capabilities, along with highly stable signal-labeling materials, to enhance the anti-interference ability and universality of the methods. (2) Deepen Digital Integration and Intelligent Analysis: The next phase of development for on-site rapid detection technologies will move beyond simple device portability towards deep digitalization and intelligence. Artificial Intelligence (AI)-assisted image analysis has become a key enabling technology, particularly for the objective interpretation of LFIA strips [137]. Algorithms based on deep learning can automatically identify weak positive bands, correct for background interference, and convert qualitative or semi-quantitative visual results into precise quantitative data, significantly improving the consistency and reliability of detection. In terms of hardware innovation, paper-based microfluidic devices, with their built-in microchannel networks, can integrate complex sample pretreatment steps (such as filtration and separation) with multiplex detection onto a single, low-cost chip, making them an ideal platform for “sample-in-answer-out” all-in-one testing [138]. Concurrently, 3D printing technology offers possibilities for rapid prototyping and customized manufacturing, applicable for producing sensor housings, microfluidic chip supports, and even functionalized electrodes tailored to specific application scenarios [139,140,141]. For continuous monitoring of aquaculture processes, developing wearable or in situ sensors that can be immersed in water bodies to enable long-term, dynamic monitoring of antibiotic concentrations is a crucial direction for early warning and residue control [142]. Ultimately, all these intelligent devices will connect to cloud-connected portable readers or smartphones, synchronizing test results, timestamps, geolocation data, and other metadata in real-time to a cloud-based regulatory platform. This will construct an interconnected monitoring network enabling data visualization, real-time risk alerts, global traceability of results, and lay the foundation for big data-driven regional risk mapping and decision support. (3) Addressing Core Challenges in Multiplex Detection: Achieving simultaneous detection of structurally diverse antibiotics is an urgent need to enhance on-site screening efficiency, but it also faces a series of scientific challenges. The primary challenge lies in balancing the specificity and breadth of recognition elements. Traditional antibodies may exhibit cross-reactivity with structural analogues, which, while beneficial for detecting drugs within the same class, can also lead to false positives. Designing broad-spectrum, high-affinity aptamers capable of efficiently recognizing multiple antibiotics with different core structures presents extreme difficulty in sequence screening and optimization. Secondly, the separation and deconvolution of signal channels are highly complex. In CRS, colors generated by different reactions may overlap and interfere; in ECRS, the oxidation-reduction peaks of different analytes may be close and hard to distinguish; in SERS, the overlapping spectra of mixtures require complex chemometric algorithms for deconvolution. Therefore, developing novel signal reporting strategies (e.g., nanolabels with distinct optical or electrical signatures) and advanced data analysis algorithms is key to accurately extracting multi-target information from complex signals. Finally, the complexity of standardization and validation increases significantly. Multiplex methods require simultaneous validation of performance for all target analytes. The workload for preparing standards, testing cross-reactivity, and conducting comparative studies against international standard methods far exceeds that of single-target methods. Establishing validation protocols widely accepted by regulatory agencies is a necessary prerequisite for promoting their practical application. 4. Focus on Industrialization Pathways: Research should consider the feasibility of large-scale production, the long-term storage stability of reagents, and the user experience design of the final product as early as possible, to bridge the gap between “excellent academic performance” and “stable, reliable products”.

## Figures and Tables

**Table 1:** Technical Parameters of Various On-site Rapid Detection.

On-Site Detection Method	Sample Matrix Type	Target Antibiotic Names	Core Technology/Core Material	Detection Limit (ppb)	Recovery Rate (%)	Precision (%)	Reference
CRS	Shrimp	KAN	hemin@Fe-MIL- “three-in-one” nanohybrids (hemin@Fe-MIL-88NH2/PtNP); exonuclease I; Aptamer	0.002	93.58–106.08	2.20–5.50	[14]
	Fish	SAs	Aptamer; Cy7	56–142	82–92	<7	[15]
	Fish, Shrimp	CAP	Aptamer; catalytic hairpin assembly; G-quadruplex	0.09	83–103.5	4.14	[16]
	Shrimp	CAP	NU-902 MOFs; Polydopamine-coated magnetic nanoparticles	0.0184	84.71–113.52	1.8–17	[17]
ELISA	Fish, Shrimp	FQs, Quinolones	Monoclonal Antibody	0.02	74.4–88.9	3.57–14.6	[18]
	Shrimp	FLU	molecularly imprinted polymer	1.09	84.2–92.3	3.3–7.0	[19]
	Frog	FQs	Broad-Spectrum Antibody	0.59	90.39–93.87	<5.11	[20]
	Fish	TC, CAP	Paper-based ELISA	0.05–0.5	/	<4	[21]
	Fish, Shellfish, Shrimp	AMOZ, AOZ, SEM, AHD, CAP, FQ	AgNPs	0.07–6.42	89.4–111.2	<10	[22]
	Shrimp	CEX	Polyclonal antibody	0.000115	72.3–95.6	4.1–22.6	[23]
	Shrimp	OTC, NFT, CAP, FQ, MG	/	1–10	/	/	[24]
	Fish, Shellfish, Shrimp	AOZ, AMOZ, AHD, SEM, CAP, FLR	carbon dots	0.0019–0.035	72.4–128.8	<25	[25]
	Shrimp	ENR; FLR	dual-template molecular imprinted polymers; QDs; Prussian blue nanoparticles	0.00133–0.00464	82.70–113.48	<11.52	[26]
SERS	Fish	NOR	Au@Ag, QuEChERS	0.0236	99.70–105.00	0.17–5.21	[27]
	Fish	CAP, TAP, FLR	Immunochromatographic test strip, Au@Ag NPs	0.20–0.78	91.5–106.4	/	[28]
	Shrimp	MG, TC	AgNPs; SPE	10	102–108	5.5–12	[29]
	Fish, Shrimp	NFT, OFX	Ti3C2Tx nanosheets; AgNPs; Nylon 66	12–35	88–107	0.3–5.5	[10]
	Fish, Shrimp	SAs	Magnetic solid phase extraction; magnetic Ti3C2Tx/Fe3O4/Ag substrate	55.9–64.0	80.2–116	0.5–5.8	[30]
	Fish, Shrimp	MG	MBs@CS@AuNPs	0.037	80.3–114.9	12.02	[31]
	Shrimp	ENR	BDT mediated-gap AuNR@Au nanoparticles	0.00012	89.2–102.4	1.70–6.38	[32]
	Fish, Shrimp	FQs	TLC, Silver sol	/	/	/	[33]
	Shrimp	NFL	Au@IS@Au	0.02	/	/	[34]
	Shrimp	TC	Aptamer-Modified Porous Anodized Aluminum Substrate; AgNPs; Electric Field Enrichment	0.000001	93.0–99.5	<5.3	[35]
ECRS	Shrimp	FZD	ZnO/ZnFe_2_O_4_	146.35	94.2–96.4	<0.21	[36]
	Shrimp	TC	Ce-MOF-808@CeO2 nanocomposite; screen-printed electrode; Aptamer	0.0000933	96.2–104	<5	[37]
	Fish, Shrimp	ENR	Ce2(MoO4)3 NPs	12.6	98.7–101	<1.8	[38]
	Shrimp	FLR	CoMnN-Cs MOF; AuNPs; Exo I; Aptamer	0.000528	97.0–100.5	2.75	[39]
	Fish, Shrimp	SA	Laser-Induced Porous Graphene Flexible Electrode; Gold Nanoshells	29.3	96.04–105.00	1.46	[40]
	Fish, Shrimp	ST	P_2_Mo_17_Co POMs; 4,4′-bipy	143.38	84.4–109.4	<5	[41]
	Shrimp	OTC	paper-based analytical device; electron-transfer resistance	0.33	/	<10	[42]
	Shrimp	CAP	α-Fe_2_O_3_	35.43	89–97	<1.8	[43]
LFIA	Fish, Shrimp	NEO, PEN, CAP	multicolor gold nanoparticles; smartphone-based detection	0.008–3.70	90–110	<0.5	[44]
	Fish, Shrimp	TC	MPF	0.045	91–114	<4.7	[45]
	Shrimp	CPAOZ	zirconium-based porphyrin MOF (PCN-222)	0.5	92.34–117.44	<11.27	[46]
	Fish, Shrimp	FQs	gold-labeled microwell	4	/	/	[47]
	Fish	β-lactam, SA, TC, quinolones, amphenicols	gold nanoparticle	0.537–2.33	87.5–115.2	<9.5	[48]
	Fish, Shrimp, Shellfish	AOZ, AMOZ, NFS	europium nanoparticles	0.013–0.023	96.0–104.4	<7.97	[49]
	Shrimp	AOZ	MnO_2_-Au NPs; colorimetric and photothermal detection	0.43	80.6–106	<0.67	[50]
	Fish, Shrimp	LMG	hyperbranched AuPBs	0.15	81–105	<3.0	[51]
	Fish	FLU	gold nanoparticle	0.39	90.35–110.15	1.89–6.91	[52]
	Shrimp	SA	PCN-222@liposome MOFs; microfluidic chip	0.01–0.1	89.65–118.78	<24.4	[53]
	Fish, Shrimp, Shellfish	AOZ, SEM, CAP, MG	2-NBA, ethyl acetate, DDB	0.01–0.80	80.0–111.3	<9.4	[54]
FRS	Fish, Crab	ENR	Aptamer; graphene oxide	5.29 (14.72 nM)	83.68–114.99	<10	[55]
	Fish, Shrimp	AHD	Aptamer; graphene oxide	0.2	89.4–110.04	1.43–14.46	[56]
	Shrimp	TCs	Heterometallic Uranyl Organic Frameworks	1060	≈100	<2	[57]
	Fish, Shrimp	MDZ	supramolecular framework Tb2.67(CB[5]2)(SIP)	27.4	95.3–107.2	<9.8	[58]
	Fish, Shrimp	RIF, RFT, RBT	Phenylboronic acid functionalized high-crystallinity fluorescent covalent organic framework (BA@COF)	30	75.20–123.46	0.09–4.46	[59]
	Shrimp	SMZ, OTC, CAP	Paper-Based Microfluidic Chip; Carbon Dot; Aptamer; MoS2 nanosheets	0.34–0.48	95.2–106.1	<6	[60]
	Fish, Shrimp	MG	Tb-MOF	3.94	99.8–107.99	<2.2	[8]
	Shrimp	TC, SMZ	carbon dots; fluorescence imprinted polymer	0.39–2.22	94–110	/	[61]
	Shrimp	CAP	zirconium-porphyrin MOF (PCN-222); Aptamer	0.00008	91.25–105.60	<5.02	[62]
	Shrimp	NFZ, NFT, FZD	donor-acceptor (D-A) type organic small molecule: P-BT3PCz; smartphone-based detection	11.45–15.10	97.63–104.53	<7.84	[63]
	Fish	NFT, NFZ, FZD, FTD	TAPA-BTD-COF	20–35	81–139	/	[64]
	Shrimp	SMZ	Fe_3_O_4_/Au/g-C_3_N_4_; Aptamer	0.87	91.6–106.82	2.8–13.44	[65]
	Shrimp	TC	Zn-BTC-BDC-NH2 MOFs;	4.89–14.37	93.62–110.66	<3.46	[66]
	Fish, Shrimp	ENR	upconverted nanoparticles; Aptamer; MIL-101(Cr) metal–organic frameworks	0.034	≈100	0.8–2.39	[67]
	Fish, Shrimp	FQs	carbon dots; smartphone-based detection	3.97	97.0–108.2	1.4–4.8	[68]

**Table 2:** Antibiotic Classification, Full Names, and Abbreviations.

Classification	Full Name	Abbreviation	Classification	Full Name	Abbreviation
Sulfonamides	Sulfathiazole	ST	Quinolones	Ciprofloxacin	CIP
	Sulfisomidine	SIM		Danofloxacin	DAN
	Sulfadiazine	SDZ		Enrofloxacin	ENR
	Sulfamethazine	SMZ		Flumequine	FLU
	Sulfamonomethoxine	SMM		Garenoxacin	GRX
	Sulfamethoxypyridazine	SMP		Levofloxacin	LEOF
	Sulfadimethoxine	SDM		Lomefloxacin	LOM
	Sulfamethoxazole	SMX		Marbofloxacin	MRF
	Sulfacetamide	SFT		Norfloxacin	NOR
	Sulfanilamide	SFL		Nalidixic acid	NALA
	Sulfapyridine	SFP		Oxolinic acid	OA
	Sulfadoxine	SFD		Ofloxacin	OFX
	Sulfaguanidine	SFG		Orbifloxacin	ORF
	Sulfachlorpyridazine	SFCP		Pefloxacin	PEFX
	Sulfamerazine	SFMZ		Rufloxacin	RUF
	Sulfameter	SFM		Sarafloxacin	SAR
	Sulfisoxazole	SIZ	β-Lactams	Amoxicillin	AMX
	Sulfaquinoxaine	SFN		Cephalexin	CEX
	Sulfaquinoxaline	SFNL		Cefuroxime	CFR
Nitrofurans	Furazolidone	FZD		Cefazolin	CFZ
	Furaltadone	FTD		Cefadroxil	CFD
	Nitrofurantoin	NFT		Penicillin	PEN
	Nitrofurazone	NFZ	Nitrofuran Metabolites	3-amino-5-(morpholin-4-ylmethyl)-1,3-oxazolidin-2-one	AMOZ
	Nitrofural	NFL		1-amino hydantoin hydrochloride	AHD
	Sodium Nifurstylenate	NFS		3-amino-2-oxazolidinone	AOZ
Macrolides	Azithromycin	AZI		3-[(4-carboxyphenyl) monomethyl] amino-2-oxazolidinone	CPAOZ
	Clarithromycin	CLR		Nitrofuran Metabolite	NFM
	Erythromycin	ERY		Semicarbazide	SEM
	Roxithromycin	ROX	Tetracyclines	Chlortetracycline	CTC
	Tylosin	TYL		Doxycycline	DOX
Amphenicols	Chloramphenicol	CAP		Oxytetracycline	OTC
	Florfenicol	FLR		Tetracycline	TC
	Florfenicol amine	FLRA	Ansamycins	Rifampicin	RIF
	Thiamphenicol	TAP		Rifapentine	RFT
Aminoglycosides	Gentamicin	GEN		Rifabutin	RBT
	Kanamycin	KAN	Diaminopyrimidines	Ormethoprim	ORM
	Neomycin	NEO		Trimethoprim	TMP
Dyes	Leucomalachite Green	LMG	Quinolone Metabolites	Deethylene-ENR	Deethylene-ENR
	Malachite green	MG	Lincosamides	Lincomycin	LCM
Glycopeptides	Vancomycin	VCM	Nitroimidazoles	Metronidazole	MDZ

**Table 3:** Situation of Antibiotic Residues in Various Aquatic Products from 2021 to 2025.

Aquatic Product Type	Antibiotic Category	Antibiotic Name	Residue Level (ppm)	Reference
Fish	Quinolones	CIP	0.00009–113.79	[71,72,73,74,75,76,77,78,79,80,81,82,83,84]
		ENR	0.000659–9.61	[72,73,74,75,76,77,78,79,80,81,83,85,86]
		OFX	0.00058–0.147	[73,79,80,87]
		OA	0.00064–0.040	[73,74,78]
		NOR	≤0.0523	[74,79,80]
		FLU	0.00003–0.0015	[74,78]
		PEFX	0.00015–0.0212	[74,80]
		SAR	0.00246–0.0036	[81,82]
		NALA	≤0.00065	[74]
		LOM	0.00082–0.00423	
		GRX	0.00907–0.03029	
		RUF	0.00170–0.00774	
		MRF	≤0.0035	[79]
		SIZ	0.02645	[88]
		SFMZ	≤0.03278	
	Sulfonamides	SDZ	0.00019–0.523	[74,78,79,89]
		SMX	0.0051–1.69	[78,79,85,88]
		SMZ	0.0011–0.3140	[73,78,79,88]
		ST	0.0021–0.0087	[78,79,82]
		SDM	0.00037–0.0014	[82,87]
		SMP	0.00075–0.024	[89,90]
		SMM	0.023	[89]
		SIM	0.002	
		SFT	0.00431	[90]
		SFCP	0.007	[78]
	Tetracyclines	DOX	0.00590–82.843	[73,78,80,81,91,92]
		OTC	0.00030–0.06	[74,78,80,82,93]
		CTC	0.00134–0.75	[74,94]
		TC	0.00083–0.0224	[80,91]
	Macrolides	CLR	0.00009–0.0435	[74,79,87]
		ERY	0.00003–0.0302	[74,81,87]
		AZI	0.00077–0.4531	[73,81]
		TYL	0.00013–0.0469	[74,79]
		ROX		
	Amphenicols	CAP	0.000792–0.00216	[80,86,95]
		TAP	0.000834–0.007	[78,96]
		FLR	0.0000615–0.192	[80,96]
		FLRA	0.000261–0.243	
	Diaminopyrimidines	TMP	0.00021–0.195	[73,74,78,79,81,97]
		ORM	0.005	[78]
	Beta-lactams	AMX	0.009–0.020	
		CFD	0.004	
		CFR	0.01151–0.07515	[98]
		CFZ	0.01521–0.03812	
	Lincosamides	LCM	0.004–0.0208	[78,79]
	Glycopeptides	VCM	0.00215–0.0216	[99,100]
	Nitroimidazoles	MDZ	0.00028–0.002	[87,101]
	Aminoglycosides	GEN	5.32	[71]
	Quinolone metabolites	Deethylene-ENR	0.00081–0.00575	[72]
Shrimp	Sulfonamides	SMM	0.065	[89]
		SMP	0.061	
		SMZ	0.0009	[102]
		SFT	0.00304–0.00307	[103]
		SMX	0.00988	
		SFL	0.00062	
		SFP	0.00021–0.00056	[90]
		SFD	0.00035–0.00144	
	Quinolones	CIP	0.0013–0.135	[102,104]
		ENR	0.002–3.683	
		OA	0.002–0.0039	[102]
		LEOF	0.0217–0.0780	[105]
		PEFX	0.00015	[104]
	Tetracyclines	OTC	0.00113–1.875	[93,102,104,106]
		CTC	0.006–0.0513	[102,104]
		DOX	≤0.0565	[104]
	Amphenicols	TAP	≤0.0413	
		CAP	≤0.0022	[95]
	Nitrofuran metabolites	SEM	0.0203–0.1157	[104,107]
Crab	Nitrofuran metabolites	SEM	0.0286–0.1528	[107]
	Diaminopyrimidines	TMP	≤0.00079	[108]
	Sulfonamides	SFG	≤0.00063	
		SFT	≤0.00161	
		SDZ	≤0.00095	
		SMZ	≤0.00111	
		SFMZ	≤0.00152	
		SFM	≤0.00437	
		SIM	≤0.00097	
		SMP	≤0.00065	
		SFCP	≤0.00392	
		SMX	≤0.00419	
		SMM	≤0.00142	
		SFD	≤0.00100	
		SIZ	≤0.00095	
		SFN	≤0.00083	
		SFNL	≤0.00646	
	Quinolones	CIP	≤0.02392	
		ENR	≤0.13571	
		ORF	≤0.00099	
		SAR	≤0.00099	
Echinoderm	Quinolones	CIP	0.0193–0.0396	[109]
		FLU	0.0012–0.0138	
		NALA	0.0011–0.0030	
		DAN	0.0604–0.0680	
	Sulfonamides	SMX	0.0080–0.0216	
Shellfish	Tetracyclines	CTC	1.19–3.068	[94]
	Amidinopenicillins	CAP	≤0.205	[95]
	Sulfonamides	SMX	0.00117–0.00126	[110]
	Diaminopyrimidines	TMP	0.0623–0.0775	
Frog	Quinolones	ENR	≤1.96	[85]

Note: The concentration ranges presented in this table are compiled as a combined range from the individual studies cited in the reference column, typically representing the reported minimum and maximum values. Unless otherwise specified in the source literature, the concentrations refer to residues detected in the edible muscle tissue of the aquatic products.

**Table 4:** Suitability and commercialization readiness of rapid detection technologies for different application scenarios.

Technology	Farm-Side Monitoring	Processing & Distribution Screening	Consumer-End Testing	Overall Commercialization Readiness
LFIA	^a^ Preferred (Mature)	^a^ Supplementary (Mature)	^a^ Viable (Mature)	High (Commercialized)
CRS	^a^ Emerging (Pre-commercial)	Potential (Pre-commercial)	^a^ Preferred (Pre-commercial)	Medium-High (Rapid translation)
ECRS	^a^ Emerging (Pre-commercial)	^a^ Primary (Commercialized)	Limited (Professional market)	Medium (Partially commercialized)
ELISA	Applicable (Commercialized)	^a^ Primary (Commercialized)	Not Applicable	High (Commercialized)
FRS	Limited (Research/Pre-commercial)	Limited (Research/Pre-commercial)	Not Applicable	Medium-Low (Prototype stage)
SERS	Long-term (Research)	Customized (Research/High-end)	Not Applicable	Low (Primarily academic)

^a^ Indicates recommended application.; terms in parentheses denote maturity status in that specific scenario: Mature, Pre-commercial, Commercialized, Research, etc.

**Table 5:** Qualitative comparison and ranking of the six on-site rapid detection technologies.

Technology	Sensitivity	Test Time	Estimated Cost per Test	Need for Pretreatment	Required Instrumentation	Technological Maturity
FRS	High	Medium (10–30 min)	Medium-High	Medium (e.g., extraction, filtration)	Dedicated reader/filter-based smartphone	Medium (Academic/emerging commercial)
LFIA	Low-Medium	Fast (<15 min)	Low	Low (Simple extraction)	None (Visual)	High (Widely commercialized)
SERS	Very High	Medium (20–40 min)	High	High (Complex, for signal enhancement)	Portable Raman spectrometer	Low-Medium (Lab/Proof-of-concept stage)
ELISA	High	Slow (1–3 h)	Medium	High (Multiple steps)	Microplate reader	High (Gold standard, well-established)
ECRS	High	Fast (<10 min)	Medium	Medium (Electrode pretreatment)	Portable potentiostat	Medium (Growing commercially)
CRS	Low-Medium	Fast (<20 min)	Low	Low (Simple extraction)	Smartphone/Visual	Medium-High (Rapidly evolving, near consumer-ready)

## Supplementary Materials

The following supporting information can be downloaded at: https://www.mdpi.com/article/10.3390/foods15071264/s1, Table S1: Database of Rapid Detection Technologies for Antibiotic Residues in Aquatic Products.

## Abbreviations

2-NBA	2-Nitrobenzaldehyde
4-MBA	4-mercaptobenzoic acid
AgNPs	silver nanoparticles
AHD	1-amino hydantoin hydrochloride
AMOZ	3-amino-5-(morpholin-4-ylmethyl)-1,3-oxazolidin-2-one
AMX	Amoxicillin
AOZ	3-amino-2-oxazolidinone
AuNP	Au nanoparticle
AuNR@Au NPs	gold nanorod@gold core–shell nanoparticles
AuNSs/LIPG	gold nanoshell-modified laser-induced porous graphene
AuPBs	Au plasmonic blackbody
AZI	Azithromycin
BDT	1,4-benzenedithiol
CAP	Chloramphenicol
CDs	carbon dots
CEX	Cephalexin
CFD	Cefadroxil
CFR	Cefuroxime
CFZ	Cefazolin
CHA	catalytic hairpin assembly
CIP	Ciprofloxacin
CLR	Clarithromycin
CM	chemical enhancement
COFs	Covalent organic frameworks
CPAOZ	3-[(4-carboxyphenyl) monomethyl] amino-2-oxazolidinone
CTC	Chlortetracycline
DAN	Danofloxacin
DDB	2,3-dichloro-5,6-dicyano-1,4-benzoquinone
Deethylene-ENR	Deethylene-ENR
DOX	Doxycycline
DRST	dissolution-recrystallization structural transformation
ELISA	Enzyme-Linked Immunosorbent Assay
EM	electromagnetic enhancement
ENR	Enrofloxacin
ERY	Erythromycin
Exo I	exonuclease I
FLR	Florfenicol
FLRA	Florfenicol amine
FLU	Flumequine
FRET	Fluorescence resonance energy transfer
FTD	Furaltadone
FZD	Furazolidone
GEN	Gentamicin
GO	graphene oxide
GRX	Garenoxacin
HPLC	high-performance liquid chromatography
HPLC-MS/MS	high-performance liquid chromatography-tandem mass spectrometry
HRP	horseradish peroxidase
IC50	half-maximal inhibitory concentration
IFE	inner filter effect
IS	internal standards
KAN	Kanamycin
LCM	Lincomycin
LEOF	Levofloxacin
LFIA	lateral flow immunoassay
LMET	ligand-to-metal energy transfer
LMG	Leucomalachite Green
LODs	low detection limits
LOM	Lomefloxacin
MBs@CS@AuNPs	chitosan-functionalized magnetic gold nanoparticles
MCP	magnetic cross-linking precipitation
MDZ	Metronidazole
MG	Malachite green
MNP@PDA	polydopamine-coated magnetic nanoparticles
MOFs	Metal–Organic Frameworks
MOFs/UOFs	Metal/heterometallic organic frameworks
MPF	Metal-polydopamine Framework
MRF	Marbofloxacin
NALA	Nalidixic acid
NEO	Neomycin
NFL	Nitrofural
NFM	Nitrofuran Metabolite
NFS	Sodium Nifurstylenate
NFT	Nitrofurantoin
NFZ	Nitrofurazone
NOR	Norfloxacin
OA	Oxolinic acid
OFX	Ofloxacin
ORF	Orbifloxacin
ORM	Ormethoprim
OTC	Oxytetracycline
PAA	Porous Anodized Aluminum
PBNPs	Prussian blue Nanoparticles
PEFX	Pefloxacin
PEN	Penicillin
PET	photoinduced electron transfer
ppt	parts-per-trillion
RBT	Rifabutin
RFT	Rifapentine
RIF	Rifampicin
ROX	Roxithromycin
RSD	relative standard deviations
RUF	Rufloxacin
SAR	Sarafloxacin
SDM	Sulfadimethoxine
SDZ	Sulfadiazine
SEM	Semicarbazide
SERS	surface-enhanced Raman scattering
SERS	Surface Enhanced Raman Scattering
SFCP	Sulfachlorpyridazine
SFD	Sulfadoxine
SFG	Sulfaguanidine
SFL	Sulfanilamide
SFM	Sulfameter
SFMZ	Sulfamerazine
SFN	Sulfaquinoxaine
SFNL	Sulfaquinoxaline
SFP	Sulfapyridine
SFT	Sulfacetamide
SIM	Sulfisomidine
SIZ	Sulfisoxazole
SMM	Sulfamonomethoxine
SMP	Sulfamethoxypyridazine
SMX	Sulfamethoxazole
SMZ	Sulfamethazine
ST	Sulfathiazole
TAP	Thiamphenicol
TC	Tetracycline
TLC	thin-layer chromatography
TMP	Trimethoprim
TYL	Tylosin
UCNPs	Upconverted Nanoparticles
UCNPs	upconversion nanoparticles
UOFs	heterometallic uranyl organic frameworks
VCM	Vancomycin
